# A double-blind randomised controlled investigation into the efficacy of Mirococept (APT070) for preventing ischaemia reperfusion injury in the kidney allograft (EMPIRIKAL): study protocol for a randomised controlled trial

**DOI:** 10.1186/s13063-017-1972-x

**Published:** 2017-06-06

**Authors:** Theodoros Kassimatis, Anass Qasem, Abdel Douiri, Elizabeth G. Ryan, Irene Rebollo-Mesa, Laura L. Nichols, Roseanna Greenlaw, Jonathon Olsburgh, Richard A. Smith, Steven H. Sacks, Martin Drage

**Affiliations:** 1 MRC Centre for Transplantation, King’s College London, Guy’s Hospital, London, UK; 2 Department of Primary Care and Public Health Sciences, King’s College London, London, UK; 3 Biostatistics and Health Informatics Department, Institute of Psychiatry, Psychology and Neuroscience, King’s College London, London, UK; 4 UCB Biopharma, Berkshire, UK; 5 Department of Transplantation, Guy’s and St Thomas’ NHS Foundation Trust, London, UK

**Keywords:** Delayed graft function, Mirococept, Complement inhibitor, Ischaemia reperfusion injury, Kidney transplantation

## Abstract

**Background:**

Delayed graft function (DGF) is traditionally defined as the requirement for dialysis during the first week after transplantation. DGF is a common complication of renal transplantation, and it negatively affects short- and long-term graft outcomes. Ischaemia reperfusion injury (IRI) is a prime contributor to the development of DGF. It is well established that complement system activation plays a pivotal role in the pathogenesis of IRI. Mirococept is a highly effective complement inhibitor that can be administered ex vivo to the donor kidney just before transplantation. Preclinical and clinical evidence suggests that Mirococept inhibits inflammatory responses that follow IRI. The EMPIRIKAL trial (REC 12/LO/1334) aims to evaluate the efficacy of Mirococept in reducing the incidence of DGF in cadaveric renal transplantation.

**Methods/design:**

EMPIRIKAL is a multicentre double-blind randomised case-control trial designed to test the superiority of Mirococept in the prevention of DGF in cadaveric renal allografts, as compared to standard cold perfusion fluid (Soltran®). Patients will be randomised to Mirococept or placebo (Pbo) and will be enrolled in cohorts of *N* = 80 with a maximum number of 7 cohorts. The first cohort will be randomised to 10 mg of Mirococept or Pbo. After the completion of each cohort, an interim analysis will be carried out in order to evaluate the dose allocation for the next cohort (possible doses: 5–25 mg). Immunosuppression therapy, antibiotic and antiviral prophylaxis will be administered as per local centre protocols. The enrolment will take approximately 24 months, and patients will be followed for 12 months. The primary endpoint is DGF, defined as the requirement for dialysis during the first week after transplantation. Secondary endpoints include duration of DGF, functional DGF, renal function at 12 months, acute rejection episodes at 6 and 12 months, primary non-function and time of hospital stay on first admission and in the first year following transplant. Safety evaluation will include the monitoring of laboratory data and the recording of all adverse events.

**Discussion:**

The EMPIRIKAL trial is the first study to evaluate the efficacy of an ex vivo administered complement inhibitor (Mirococept) in preventing DGF in cadaveric human renal transplantation. Mirococept has a unique ‘cytotopic’ property that permits its retention in the organ microvasculature.

**Trial registration:**

ISRCTN registry, ISRCTN49958194. Registered on 3 August 2012.

**Electronic supplementary material:**

The online version of this article (doi:10.1186/s13063-017-1972-x) contains supplementary material, which is available to authorized users.

## Background

Ischaemia reperfusion injury (IRI) is a consequence of kidney transplantation that often progresses to the clinical diagnosis of delayed graft function (DGF). DGF has traditionally been defined as the requirement for dialysis within the first week after transplantation, although more than 10 definitions have been recorded in the literature [1]. Approximately one third of all kidney transplants will develop DGF with this proportion rising to 50% when the organ is retrieved by donation after circulatory death (DCD) [2]. DGF is associated with higher rates of acute rejection and with reduced long-term kidney survival even in patients who did not undergo rejection [3]. In this regard, strategies to prevent IRI and the subsequent development of DGF are of paramount importance for improving renal graft outcomes.

The reperfusion of the ischaemic kidney induces a proinflammatory reaction in which activation of the complement system plays a central role [4]. Complement-depleted or deficient animals exhibit reduced post ischaemic acute renal failure and chronic nephropathy [4–6]. Recent studies have indicated that locally synthesised complement components play a more important role in activating local inflammatory processes and mediating graft immunogenicity than complement components from the systemic circulation [7]. The central step in complement activation is the cleavage of C3 to C3b by C3 convertase. Membrane-bound C3b binds to activated factor B and forms the C3 alternative pathway convertase enzyme, resulting in further C3 cleavage to C3b. The primary trigger is now believed to be the lectin pathway, followed by amplification through the alternative pathway, both pathways converging on the central component C3 [8]. This ultimately leads to C5a- and C5b-9-mediated injury of the renal tubule, and C3a- and C5a-mediated enhancement of the recipient immune response [9, 10]. In vivo protection against aberrant complement activation is prevented by the natural regulators of complement activation (RCAs), which can be membrane bound (e.g. CD35 — also known as CR1 — CD46 and CD55) or soluble (e.g. factor H). RCAs act via two mechanisms: one by inhibiting C3 and C5 convertases by accelerating their decay and the other by acting as cofactors for factor I-mediated proteolysis of C3b/C4b to the inactive iC3b and C3d and C4d respectively [11]. Microarray analyses of human renal graft biopsies before transplantation have shown a negative correlation between complement gene expression and graft function in the early (2–3 days) and late (2–3 years) post-transplant period [12]. Of note, the duration of cold ischaemia was positively associated with the level of complement gene expression. This relationship was previously demonstrated in a mouse renal isograft model where intrarenal C3 mRNA level increased in relation to the duration of cold ischaemia time (CIT) [13]. Intriguingly, in this model, local C3 — mostly produced by the tubular epithelium — was essential for complement-mediated reperfusion damage [13].

Together, these mechanisms provide a rationale for local therapeutic manipulation of C3 function during early transplantation in order to improve the short- and possibly longer-term outcomes of renal grafts. The local control of complement activation would have the advantage of providing graft protection whilst avoiding the systemic abrogation of the complement system and the consequent impairment of recipient immune defences.

Mirococept (APT070) is a highly effective complement inhibitor which is derived from human CR1 (CD35). It consists of three functional units: the terminal three domains of CR1 which contain its biological activities, a membrane-associating peptide and a membrane-inserting myristoyl group [14]. The last two units permit the binding to and insertion into the cell membrane. Mirococept is unique in that it is engineered to bind to cell surfaces in bulk [15] and can block the complement system at the C3 level. It does not inhibit proteases generally, and its action is restricted to the complement system.

Mirococept has been shown to inhibit complement activation and the subsequent inflammatory reaction in a variety of experimental diseases that are associated with complement activation such as rheumatoid arthritis [16], Guillain-Barre syndrome [17], intestinal ischaemia [18], myocardial ischaemia [19] and kidney transplantation [6, 20]. We previously developed a strategy to administer Mirococept in the donor kidney, thereby avoiding any undesirable consequences from systemic complement inhibition [6]. After being administered to the kidney ex vivo, via the renal artery, Mirococept localises to the graft endothelial and epithelial cells. In a rat transplant IRI model we showed that local inhibition of complement activation within the graft by Mirococept reduced complement-mediated injury [6]. Specifically, Mirococept reduced inflammatory injury and allograft rejection, thus improving graft function in the short (throughout the first week) and long term (20 weeks). As these results were obtained after the relatively short ischaemia time of 30 min, we further evaluated Mirococept efficacy in a rat renal transplantation model of prolonged cold ischaemia (16 h). Similarly to our previous findings, Mirococept reduced ischaemia reperfusion damage and inflammation by inhibiting complement activation at the C3 level [20]. It also increased the number of donor organs that survived after transplantation by at least twofold.

The safety of Mirococept has been documented by preclinical, phase I and phase IIa clinical studies (see section on Safety evaluation and recording of adverse events). Moreover, in the phase IIa clinical trial where Mirococept or placebo (Pbo) was administered to the kidney ex vivo before transplantation, a trend was observed toward lower creatinine in the Mirococept group [21]. The aim of the current trial is to provide further evidence supporting the efficacy of Mirococept in preventing DGF in cadaveric human renal transplantation.

## Methods/design

### Trial design

This is a multicentre, double-blind, randomised case-control trial, designed to test the superiority of Mirococept in the prevention of DGF in cadaveric renal allografts, as compared to standard cold perfusion fluid (Soltran®). Patients will be randomised to treatment or Pbo, and randomisation will be carried out in blocks and stratified by centre, type of donor, i.e. Donation after Circulatory Death (DCD)/Donation after Brain Death (DBD), and whether the organ has been machine pumped. Immunosuppression therapy, antibiotic and antiviral prophylaxis will be administered as per local centre protocols. In order to estimate the dose-response curve and obtain the minimum effective dose (MED), we will use an adaptive design known as the cumulative cohort design (CCD) for dose finding, first described by Ivanova et al. [22, 23]. In most cases this adaptive design is more powerful and effective in finding differences against Pbo and in estimating the dose-response curve, compared to an equal allocation design. This is because the adaptive procedure successfully assigns more patients to Pbo and the target dose, i.e. the MED. Reporting conforms to the Standard Protocol Items: Recommendations for Interventional Trials (SPIRIT) checklist (see Additional file 1).

### Selection of participants, recruiting and consent

**Fig. 1:**
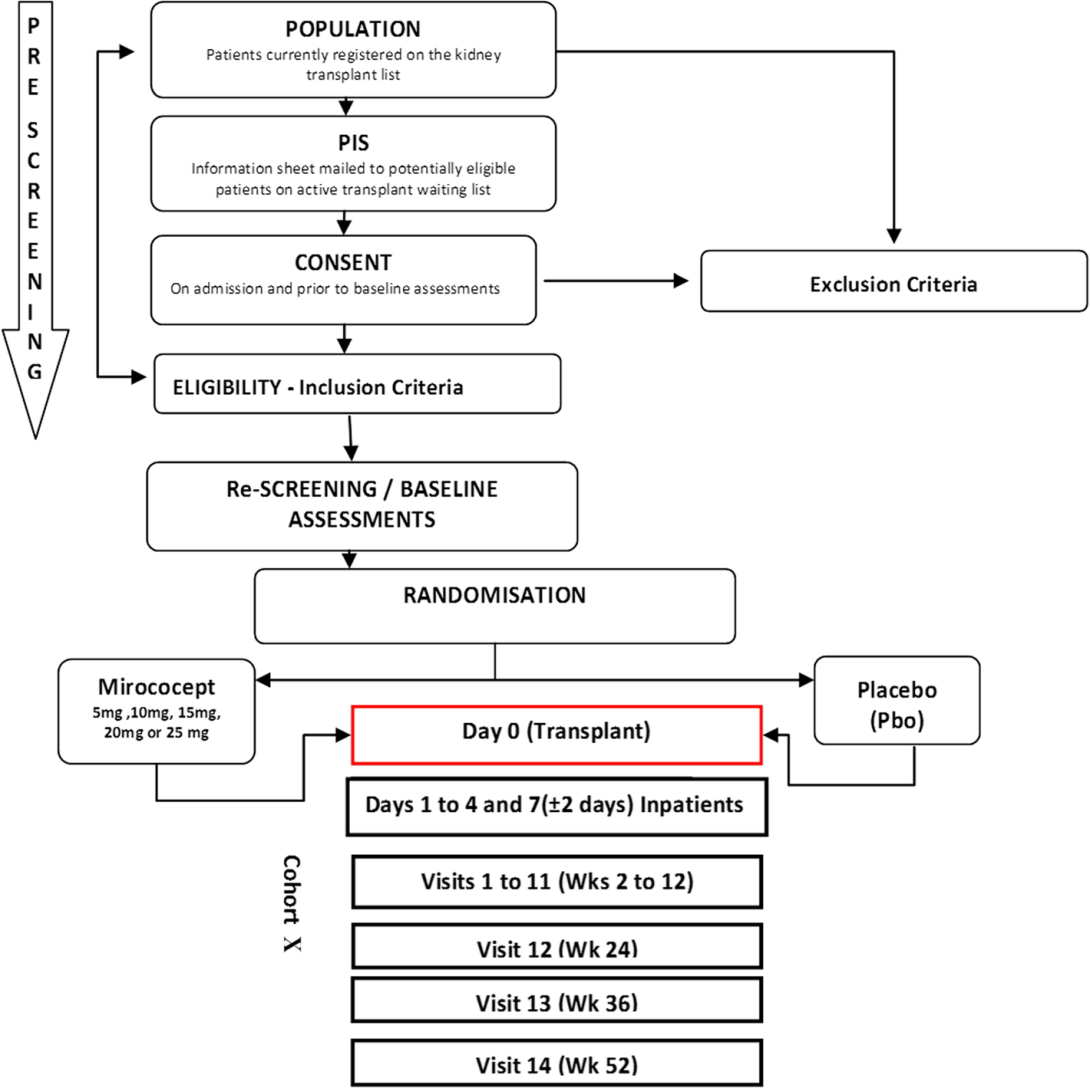
Study flowchart

The inclusion and exclusion criteria are shown in Table 1. Potential participants will be identified from the active kidney transplant waiting list. After initial screening, eligible participants will be contacted and Patient Information Sheet part A (see Additional file 2 for template copies of the Patient Information Sheet) mailed to the patient’s home address. Participants will be able to discuss the trial with their recruiting site physician and decide whether they want to participate when a donor kidney becomes available. At the time of transplant, all patients will be screened for eligibility on arrival as part of their routine assessment for transplantation. Eligible participants will be given Patient Information Sheet part B and consented on admission (see Additional file 3 for a template copy of the Informed Consent Form) (see Fig. 1: study flowchart).

**Table 1:** Inclusion and exclusion criteria

Inclusion criteria	1. Patient must be 16 years of age or older and registered on the kidney transplant list. 2. Patient must be willing to participate in the study and provide written informed consent. 3. Patient must have the ability to comply with the study requirements. 4. Donor must be older than 10 years of age. 5. Patient is on dialysis.
Exclusion criteria	1. Patient is recipient of a living-donor kidney. 2. Patient is a recipient of a DCD kidney Maastricht category 1 or 2. 3. Patient has evidence of current infection with HIV, HBV or HCV. 4. Patient is recipient of a paediatric en bloc or a adult double renal transplant. 5. Any recipient of a multi-organ transplant or a previous recipient of a non-renal solid organ transplant. 6. Females who are pregnant or lactating. 7. Male and female patients not willing to use contraception for at least one month post-transplant. 8. Any planned ABO blood group or HLA antibody-incompatible transplant. 9. Patient is involved in other experimental drug trials.

### Recruitment in cohorts

**Fig. 2:**
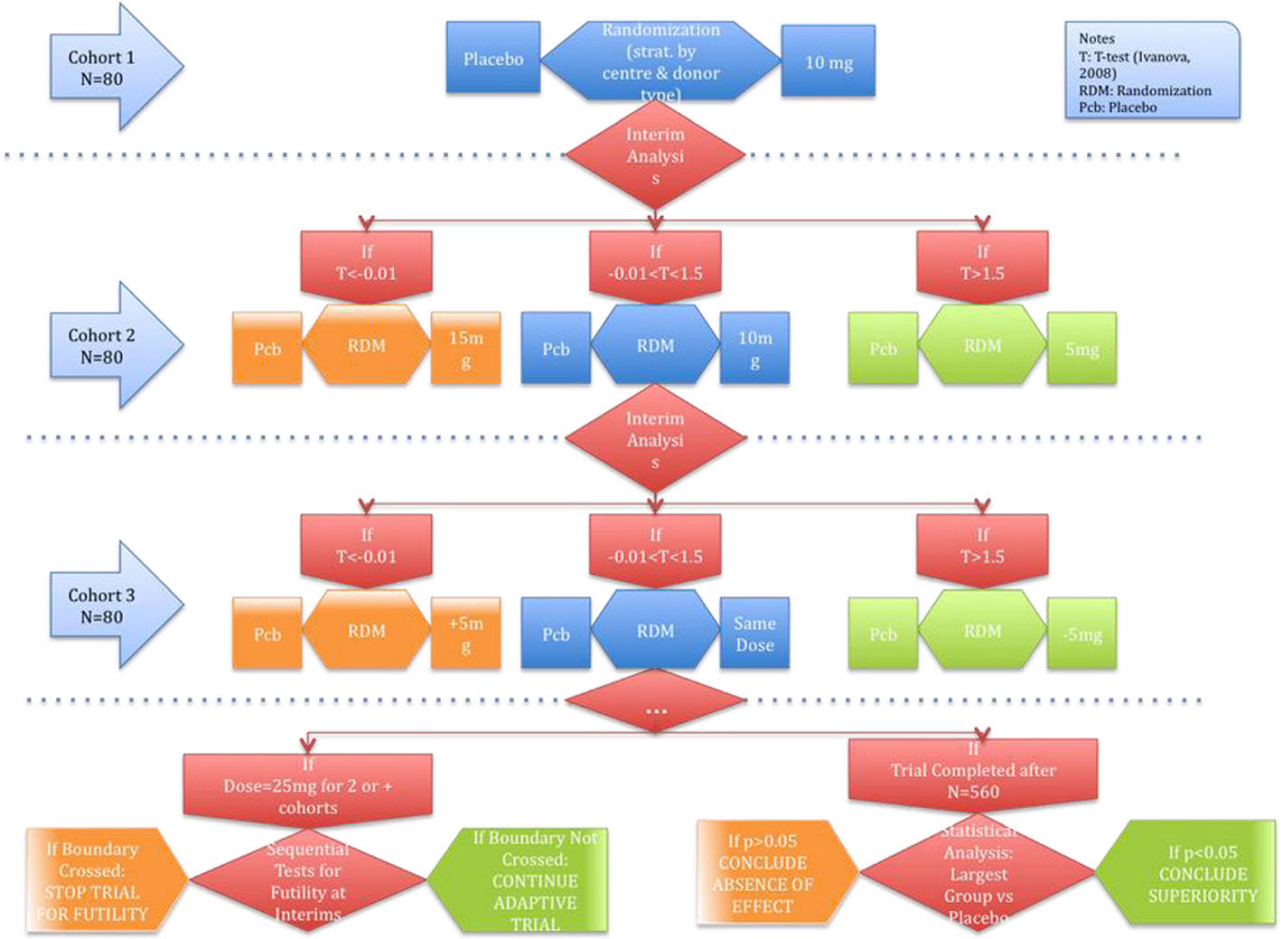
Recruitment cohorts

Patients will be enrolled in cohorts of *N* = 80 with a maximum number of 7 cohorts (maximum number of transplants = 560). Each cohort will be randomised to one of two groups, Pbo or one dose of Mirococept. As originally planned, the first cohort will be randomised to Pbo or 10 mg of Mirococept. After the completion of each cohort, an interim analysis will be carried out in order to evaluate the dose allocation for the next cohort (Fig. 2: recruitment cohorts). Based on the data from previous studies, we will work with five possible doses: 5 mg, 10 mg, 15 mg, 20 mg and 25 mg.

### Randomisation

Recruiting site personnel will be responsible for randomisation of individual patients through the described process. Patients will be randomised to treatment or Pbo with an allocation ratio of (Pbo:drug) 1:2 (i.e. 27 Pbo/53 drug per cohort). Randomisation uses minimisation for the following factors:

- □ Centre
- □ Donation after Circulatory Death (DCD) or Donation after Brain Death (DBD)
- □ Machine pump use

Once ensuring all inclusion and none of the exclusion criteria are met and written informed consent is obtained, the patient must be registered on the MACRO trial database and is assigned a unique patient identifier. The patient can then be randomised, with this patient ID, via SealedEnvelope, an independent web-based company that will be accessible to all the centres via the Internet (https://www.sealedenvelope.com/empirikal/). In the event that a pack number is assigned, but the transplant does not go ahead, the pack is quarantined, in the area predesignated for quarantined kits, until it can be reassigned for use.

### Preparation of the kidney and administration of Mirococept

The following steps are used to prepare the kidney and administer Mirococept:

1. Using a 1000-ml Soltran bag, the first 500 ml will be used to flush the organ prior to administration of the investigational medicinal product (IMP). (N.B University of Wisconsin (UW) solution will affect the bioavailability of Mirococept and is therefore not used.)
2. Machine-pumped organs must not to be placed back on the pump after initial flush with Soltran has been completed.
3. The kidney will be placed in a container of ice slush for bench work.
4. The vials of IMP take approximately 15 min to thaw, and so should be removed from the freezer with sufficient time to allow for this.
5. Whilst the kidney is in ice slush, all five vials of the IMP are drawn up from the patient’s allocated drug kit and added to the remaining 500 ml Soltran (500 ml should be left in the 1000-ml bag following the initial use of 500 ml for the flush). Once the IMP is mixed with the remaining 500 ml of Soltran in the bag, the fluid is then perfused through the kidney via the renal artery (arteries), under 1 m hydrostatic pressure.
6. The perfusion will be conducted via a cannula held in place manually such that as much perfusion solution as possible drains through the kidney.
7. If there is more than one renal artery, and they are of sufficient size, the IMP should be distributed through each of the arteries according to their estimated proportion of the kidney that they vascularise.
8. The administration of IMP should take approximately 15 min.
9. Following perfusion the cannula can be removed and the kidney repacked for cold storage.
10. No post-perfusion flush or arterial or renal vein clamping will be required.

The transplantation procedure will continue in the standard manner, and the patient will be monitored according to standard procedures.

At the Guy’s site only, two biopsy samples will be taken (one half of each core that is taken routinely as part of clinical practice will be used in research): before perfusion with IMP and blood and after perfusion with IMP and blood. The research biopsies will be used to determine the presence of CR1, C3d and membrane attack complex (MAC) deposition. This information will not be available to the clinician, so as to maintain blinding.

### Concomitant medication

Patients in this trial will receive the local standard treatment for those undergoing a renal transplant. This encompasses presurgery medication, general anaesthesia, recovery from anaesthesia, pain control medication and anti-infective therapy (viral, fungal and bacterial). Immunosuppression therapy should be given as per local standard protocol.

Anaesthesia-related medication will not be regarded as concomitant medication.

Other treatments which will not be considered as concomitant medication are:

- □ Pneumocystis jiroveci pneumonia (PJP, PCP) prophylaxis
- □ Cytomegalovirus (CMV) prophylaxis
- □ Tuberculosis (TB) prophylaxis

The use of other medications will be recorded in detail in the patient study MACRO database. Experimental drug therapies will not be allowed for the duration of the trial.

### Research samples

All laboratory investigations that are required are part of the routine management of renal transplant patients, except the following which are additional:

- □ Serum samples for antibodies to Mirococept
- □ Serum samples for Mirococept (*at the Guy’s site only*)
- □ Serum samples for complement activity
- □ Whole blood samples for RNA bio-markers (*cohort 1 patients only*)
- □ Plasma samples for symmetric dimethylarginine (SDMA) levels
- □ Urine samples for markers of tubular damage
- □ Urine samples for C3a
- □ Urine samples for RNA bio-markers (*at the Guy’s site cohort 1 patients only*)
- □ Renal biopsy tissue for research (*at the Guy’s site only*)

Although serum, plasma and RNA do not fall under the Human Tissue Act (HTAct) relevant material, all samples will be processed, tracked and stored according to HTAct 2004 and the European Union Directive Guidelines. The EMPIRIKAL Laboratory Manual gives full details on collection, processing, storage and shipment of research samples (see Additional file 4).

The trial’s assessment schedule is shown in Table 2.

**Table 2:**
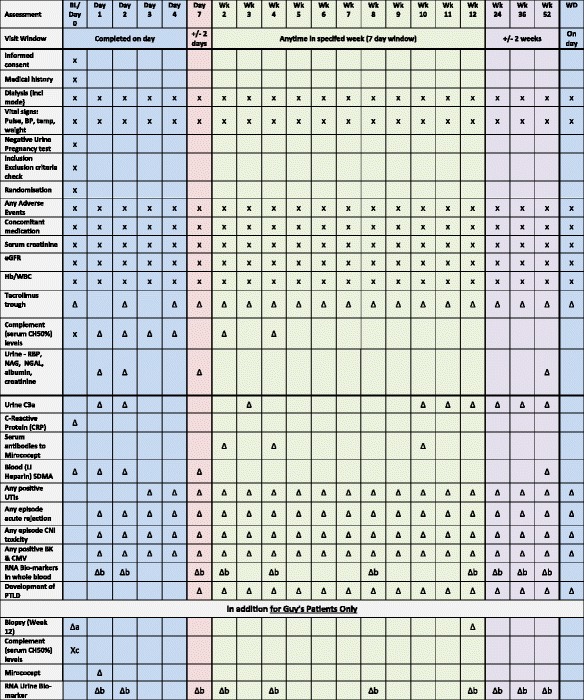
Assessment schedule

Key

Where an ’x’ is contained within a field, this denotes that the associated data is essential and if missed will be classified as a protocol deviation

Where a ’Δ’ is contained within a field, this denotes that the associated data is desirable, and whilst every effort should be made to record these data, missing data will *not* constitute a protocol deviation

*eGFR* estimated glomerular filtration rate, *Hb* haemoglobin, *WBC* white blood cells, *RBP* retinol binding protein, *NAG N*-acetyl-β-d-glucosaminidase, *NGAL* neutrophil gelatinase-associated lipocalin, *CNI* calcineurin inhibitor, *PTLD*, post-transplant lymphoproliferative disorder

### Outcome measurements

#### Primary outcome

The primary outcome is DGF, defined as the requirement for dialysis during the first week after transplantation.

#### Secondary outcomes

The secondary outcomes are as follows:

1. Duration of DGF
2. Mean calculated glomerular filtration rate (GFR) (MDRD) at 12 months
3. Mean calculated GFR (Cockcroft-Gault) at 12 months
4. Functional DGF, i.e. the absence of a decrease in serum creatinine of at least 10% per day for at least 3 consecutive days in the first week post-transplant (not including patients with biopsy-proven acute rejection (BPAR) or calcineurin inhibitor (CNI) toxicity within first week)
5. Primary non-function, defined as a permanent lack of function of the allograft from the time of transplantation
6. Area under the curve (AUC) of the daily serum creatinine days 1–14
7. Biopsy-proven acute rejection at 6 months and 12 months including borderline changes
8. CNI toxicity
9. Time of recipient hospital stay on first admission
10. Time of recipient hospital stay in first year
11. Graft 1-year survival (censored and uncensored for death of patient with functioning allograft)
12. Recipient 1-year survival

### Code break

Randomisation data are kept strictly confidential, accessible only to authorised persons not involved in any aspect of the conduct of the trial, until the time of unblinding. At every interim review and at the conclusion of the trial, the occurrence of any emergency code breaks will be determined after return of all code break reports to King’s Health Partners Clinical Trials Office (KHP-CTO). When each cohort has been completed, the data file verified and protocol violations determined, the medication randomisation codes will be broken and made available for interim data analysis.

A 24-h medical information and emergency unblinding service will be provided by the Emergency Scientific Medical Service (ESMS). Code break envelopes will be provided to ESMS. If a request for unblinding is received by ESMS, they will notify the KHP-CTO by fax on the next working day, including information on whether the subject was unblinded or not. Only requests to unblind from a medical doctor will be accepted (e.g. accident and emergency doctor, general practitioner). The KHP-CTO will inform the Chief Investigator (CI) Team and recruiting Principal Investigator (PI) of an unblinding request where appropriate. If the participant has been unblinded, the recruiting PI and CI may not be informed of the treatment allocation unless that information is needed for the participant’s medical care. The decision on whether to inform the recruiting PI will be made by the CI in conjunction with the Trial Statistician. All trial subjects will be issued with a card containing trial identification information and emergency unblinding/medical information contact details.

### Withdrawal of subjects

The subjects will be advised in the Patient Information Sheets and during the consent process that they have the right to withdraw from the study at any time without prejudice, and may be withdrawn at the discretion of the Investigator at any time. In the event that a subject drops out of the trial or is withdrawn from the trial, the appropriate withdrawal electronic Case Report Form (eCRF) will be completed on the MACRO database (if applicable, blood samples should be taken for analysis). Reasonable effort will be made to contact any subject lost to follow up during the course of the trial in order to complete assessments and retrieve any outstanding data. The cases of patients who die during the trial will be assessed by the external data monitoring committee as soon as possible to determine whether the trial should be halted. If the cause of death of a patient is deemed unrelated to the trial, that patient may be replaced in the analysis by another eligible patient.

### Expected duration of trial: end of study definition

Patient recruitment is expected to take approximately 24 months (including interim reviews), and follow-up will continue until the last recruited patient completes their 12 months follow-up unless the trial is terminated earlier for futility. The trial will be closed when all participants have made their final follow-up visit, the data are entered into the database and all queries resolved and the database locked. All serious adverse events (SAEs) will be followed up for a further 30 days after the stopping of the trial (last patient’s final 1 year follow-up visit date) or until resolution.

### Safety evaluation and reporting of adverse events

The Investigator or clinician with delegated authority will review all clinical laboratory reports and will sign and date them on the day of review. If there are any findings outside the normal range, the investigator will confirm whether the result is clinically significant or not. Any laboratory report indicated as being clinically significant will be recorded on the Adverse Event section of the CRF.

All adverse events will be recorded from time of consent. However, as Mirococept is administered to the kidney ex vivo and is cytotopic, i.e. cell membrane associated, very little is expected to enter the systemic circulation. The safety of Mirococept has been demonstrated in preclinical studies as well as in phase I and II clinical trials. In the phase I randomised, double-blind, placebo-controlled, dose escalation study, doses of up to 100 mg given to healthy men as a single intravenous (IV) infusion were tolerated well. There were seven dose levels, 2, 5, 10, 20, 40, 70 and 100 mg, and six subjects participated in each dose level, four subjects receiving active drug and 2 receiving Pbo. There were no deaths or SAEs and the number of treatment-related AEs was low up until the 100 mg Mirococept dose. With 100 mg IV infusion there were increased events in three out of four subjects receiving the active medication. For this dose level, the most common complaints were general disorders (fatigue, headache) and nausea with one occurrence of pyrexia. The lack of any consistent pattern in the AEs for doses lower than 100 mg Mirococept indicated good tolerability. There were no treatment-related changes in vital signs or clinical laboratory parameters. The plasma elimination half-life in healthy subjects was approximately 3 h, and for doses below 10 mg there was no apparent inhibition of systemic complement activation. A pilot phase II study conducted in 12 patients using a dose of 10 mg of Mirococept perfused into donor human kidneys which were then transplanted also showed that this agent and mode of administration was well tolerated. More than 80% of the drug was retained in the graft and no inhibition of circulatory complement was detected. The starting dose level of 10 mg was based on animal data [6, 20] and was found to be safe in phase I and pilot phase IIa studies [21].

In the present protocol, we do not expect more than approximately 20% of the maximum dose administered to the graft to reach the systemic circulation. At the doses proposed (5–25 mg), this exposure lies in the range of 1–5 mg of drug. Therefore, we do not consider drug-related AEs to be likely.

### Statistical analysis

#### Basic power calculation

We will consider the use of Mirococept superior to standard cold perfusion if the upper boundary of the 95% confidence interval for the absolute DGF risk difference includes the minimum considered clinically significant (10%). All calculations have been made presuming an absolute risk of DGF of 35% [2] and thus expecting it to drop to at least 25% in the treatment group.

Using an asymmetric two-sided group sequential design, with 80% power and 5% type I error [24], the maximum sample size is 283 transplant pairs, a total of 566 patients. Based on this sample size calculation, we used a total sample size of 560 to perform a simulation study under different scenarios of dose response to optimise the performance of the adaptive design and determine the dose allocation rules (results given in Additional file 5). The power to detect differences in the response between the accumulated Pbo patients and the dose to which the largest number of patients were allocated was above 0.80 for three out of the four scenarios of dose response, where the fourth scenario was one where there was no effect. Under the absence of an effect, only 5% of the simulated trials found a significant effect, which is consistent with a 5% type I error.

#### Interim analysis and dose allocation

As discussed earlier, patients will be recruited in cohorts of *N* = 80 with a maximum number of 7 cohorts. Five possible doses will be assessed: 5 mg, 10 mg, 15 mg, 20 mg and 25 mg. The adaptive allocation will be based on a *t* test-like statistic that compares the difference between response (accumulated) at the current dose with the mean response at Pbo plus our target difference 0.10 [23]. In brief, the current dose is repeated if the current estimated difference in responses between dose and Pbo (scaled by the variance) is close to the target 0.10, and changed if otherwise (Fig. 2: recruitment cohorts). Simulation studies (Additional file 5) showed that the following allocation rules provided optimal performance:

- *If t statistic < –0.01*: *Increase by one dose (e.g. from 10 mg to 15 mg)*
- *If –0.01 < t statistic < 1.5*: *Repeat the current dose (e.g. keep 10 mg)*
- *If t statistic > 1.5*: *Decrease by one dose (e.g. from 10 mg to 5 mg)*

Note that a *t* statistic = 0 implies a difference in the rate of DGF between the drug and Pbo groups of 0.10, the minimum clinically significant; a positive *t* statistic implies that the difference in DGF rate between Pbo and the drug is larger than 0.10, and a negative *t* statistic implies that the difference is lower than 0.10.

### Final data analysis

The main analysis of the primary endpoint will consist of a logistic (binary) regression to examine whether the use of Mirococept as compared to standard cold storage, in the context of other relevant covariates, influences the risk of DGF. The same procedure will be applied to binary secondary endpoints. A linear regression will be used for secondary endpoints measured on a continuous scale (e.g. GFR), using a log transformation when the distribution deviates from normality or for time measures (e.g. duration of DGF). For the analysis of graft survival, the comparison between the two groups will be carried out using hazard ratios calculated from a Cox proportional-hazards model, adjusted by the relevant covariates.

### Handling of missing data in secondary outcomes

It is expected that approximately 10% of patients could be lost to follow-up, due to drop-out or transfer to other centres. For time-to-event outcomes, survival analysis will be used, and patients lost to follow-up will be classified as censored. For binary and continuous endpoints we will first check whether patients lost to follow-up differ significantly from the rest in their clinical characteristics, expecting absence of differences. Subsequently, the analyses will first be performed with the available data and then be repeated after carrying out imputation of the data missing due to loss at follow-up. This may add power to the statistical test, but the direction of the effect should not differ significantly from that observed without imputation.

The study may also be stopped for futility before the maximum sample size is reached. Simulation results show that, in the absence of effect, the adaptive allocation reaches the maximum dose quickly. *p*-value cut-offs to stop for futility will be applied starting from the interim analysis after two consecutive cohorts on the maximum dose (25 mg).

## Discussion

In the last two decades the incidence of DGF has significantly increased, probably due to the use of expanded criteria donors (ECDs) and DCD donors [2]. As discussed earlier, this common complication of renal transplantation is associated with detrimental effects on both short- and long-term graft outcomes as it is linked to increased rates of acute rejection and chronic allograft nephropathy. To date, there is no established pathway-specific approach for the prevention and treatment of DGF. Over the last decade, considerable progress has been made in the understanding of the molecular mechanisms implicated in the pathogenesis of acute kidney injury that frequently leads to DGF [1]. In this regard, various interventions for the prevention and treatment of DGF are currently under evaluation in the preclinical and clinical settings. These interventions can be divided into organ-directed techniques (e.g. machine perfusion of the donor kidney, use of different preservation solutions) and recipient-directed treatments. Recipient-directed treatments include the ischaemic preconditioning of the recipient, vasodilatory agents, anti-inflammatory and anti-apoptotic agents and different induction immunosuppression regimens [1]. However, the administration of these compounds in the recipient’s systemic circulation would entail the risk of serious adverse reactions. In contrast, kidney delivery techniques have the advantage of selectively inhibiting the local inflammatory processes of IRI, and at the same time they minimise the risk of systemic side effects.

To our knowledge, this is the first clinical trial to implement this innovative method of ex vivo administration of a cytotopic complement inhibitor (Mirococept) in renal transplantation.

## Trial status

The first participant was randomised on 29 October 2015. At the time of manuscript submission, recruitment for cohort 1 has been completed. The target is for completion of enrolment in October 2017 or as soon as possible thereafter.

## Additional files

Additional file 1: Spirit checklist. (DOC 122 kb)

Additional file 2: Patient Information Sheet part A. (DOC 61 kb)

Additional file 3: Informed Consent Form. (DOC 57 kb)

Additional file 4: EMPIRIKAL Lab Manual. (PDF 1263 kb)

Additional file 5: Simulation study. (DOC 231 kb)

Additional file 6: Data Management Plan. (DOC 450 kb)

## Abbreviations

APT070: Mirococept
BPAR: Biopsy-proven acute rejection
CIT: Cold ischaemia time
CMV: Cytomegalovirus
CNI: Calcineurin inhibitor
CR1: Complement receptor 1
CRF: Case Report Form
CRP: C-reactive protein
DBD: Donation after Brain Death
DCD: Donation after Circulatory Death
DGF: Delayed graft function
eGFR: Estimated glomerular filtration rate
HBV: Hepatitis B virus
HCV: Hepatitis C virus
HIV: Human immunodeficiency virus
HLA: Human leukocyte antigen
IMP: Investigational medicinal product
IRI: Ischaemia reperfusion injury
KHP-CTO: King’s Health Partners Clinical Trials Office
MAC: Membrane attack complex
MDRD: Mean calculated GFR
MHRA: Medicines and Healthcare products Regulatory Agency
MRC: Medical Research Council
NAG: *N*-acetyl-β-d-glucosaminidase
NGAL: Neutrophil gelatinase-associated lipocalin
Pbo: Placebo
PTLD: Post-transplant lymphoproliferative disorder
RBP: Retinol binding protein
RCA: Regulators of complement activation
REC: Research Ethics Committee
SDMA: Symmetric dimethylarginine
SPC: Summary of Product Characteristics
UTI: Urinary tract infection
