# Supplementary material for: A double-blind randomised controlled investigation into the efficacy of Mirococept (APT070) for preventing ischaemia reperfusion injury in the kidney allograft (EMPIRIKAL): study protocol for a randomised controlled trial
Source: Trials. 2017 Jun 6;18:255. doi: 10.1186/s13063-017-1972-x (PMC5461672; doi:10.1186/s13063-017-1972-x)
Supplement: Supplementary file 2 — Patient Information Sheet part A. (DOC 61 kb) [file 13063_2017_1972_MOESM2_ESM.doc]

**[To be printed on hospital headed paper]**

**Participant Information Leaflet**

**for Study on Kidney Transplants**

**STUDY TITLE**

**An Investigation into the Efficacy of Mirococept for Preventing Ischaemia-reperfusion Injury in the Renal Allograft (EMPIRIKAL)**

**Working Title**

Treatment of the donor kidney with Mirococept to see if this improves the recovery of the kidney after transplantation.

**INVITATION**

This leaflet is to inform you about a clinical research study which will involve treating the donor kidney with Mirococept (active drug) and (Soltran®) in comparison to standard cold perfusion fluid (Soltran®) prior to transplantation of the organ.

Before you decide on whether you are willing to take part it is important for you to understand why the research is being done, what it will involve and how long you will need to participate in the study. Kindly take time to read the following information carefully and discuss it with friends, relatives or your hospital doctor if you wish. Please ask us if there is anything that is not clear or if you would like more information. Take time to decide whether or not you wish to take part when a donor kidney becomes available.

We will ask you if you want to voluntarily take part before your transplant. A full study Patient Information Sheet (PART B) and Informed Consent form will be provided to you should you wish to take part.

This study does not affect your standard transplant treatment.

Thank you for reading this.

**WHAT IS THE PURPOSE OF THE STUDY?**

Before a kidney can be transplanted into a recipient it has to be removed from the donor and transported to the hospital of the recipient. During this period the kidney does not have a blood supply and is stored in a cold solution to minimise damage caused by lack of blood supply.

One of the factors that can contribute to the damage of the kidney can potentially be blocked by a drug called Mirococept. Mirococept has been used before in 28 healthy volunteers and in 12 patients undergoing kidney transplantation. At the dose range proposed in this trial there were no described side effects.

Mirococept is a modified form of a human protein whose primary biological and pharmacological activity is to block the tissue damage caused by the uncontrolled activation of the complement system (a component of the human immune system) which occurs during kidney transplantation. Mirococept restores a natural control system that is lost during the process of transfering an organ from the donor to the recipient of a transplant.  FUJIFILM Diosynth Biotechnologies UK Limited (formally Avecia Ltd) are the manufacturers for Mirococept and have made this drug previously for clinical trials.

The aim of this study is to give this Mirococept and Soltran® to the kidney, after the kidney has been removed from the donor and before it is transplanted into the recipient. The aim of the study is to find out whether damage to the kidney can be prevented and to see if the new treatment improves the function of the kidney and whether this might extend the life of the kidney.

This is a placebo-controlled study. This means that the donor kidney would be treated with either the active drug (Mirococept and Soltran®) or a standard treatment (Soltran® alone). In total, there will be 560 participants in the study - 370 will receive kidneys treated with the active drug and the remaining 190 participants with the standard treatment. The study will take place across the UK.

**WHY HAVE I BEEN INVITED?**

You have been invited to take part in this study because you are registered on the kidney **transplant waiting list** and currently are waiting to receive a donor kidney. Your kidney team have assessed you to be suitable to potentially take part in this study.

**DO I HAVE TO TAKE PART?**

Your participation is entirely voluntary. If you do decide to take part, we will give you this information sheet to keep and you will be asked to sign a consent form. If you decide to take part you are still free to withdraw at any time and without giving a reason. This will not affect the standard of care that you receive.

**WHAT WILL HAPPEN TO ME IF I TAKE PART?**

If you agree to take part, please let us know beforehand if you have been involved in any other research study during the last year. If you have we will discuss with you and your clinical team whether you are allowed to participate in more than one clinical study.

All participants will receive standard treatment with drugs to prevent rejection of the kidney transplant. In addition, some of the participants will receive a donor kidney that has already been treated with the study medication. The other patients will receive a donor kidney that has been treated with the standard substance. Neither the person who gives the treatment into the kidney nor the person who receives the kidney will know which of the kidneys have been treated with the active and standard medicines. This will be decided by a computer in a random way, like the toss of a coin.

To follow up the effect of this treatment on the kidney we will need to take some blood samples and urine samples during your routine outpatient clinic visits during the first year. This will be over and above your routine blood samples. Depending on which hospital you are treated at we would ask for up to a total of 14 blood samples, in the first year after your transplant, each consisting of 20mL (this is about an egg-cup full). These will be taken at the following time points; days 1 to 4 and weeks 1, 2, 4, 8, 10, 12, 24, 36 and 52. An unscheduled sample may be requested should you choose withdraw from the study at any point.

In addition, depending on the hospital you are being treated at we will also ask for up to 13 urine samples, each consisting of 50mL. These will be taken at the following time points days 1, 2, 7 and weeks 2, 3, 4, 8, 10, 11, 12, 24, 36 and 52. An unscheduled sample may be requested should you choose withdraw from the study at any point.

On month 3 after the transplant we might ask some people to consider having a biopsy of the kidney transplant. The biopsy will be helpful to assess your progress, but if you decide that you do not want the biopsy you can still be part of the study.

Some of the blood, urine or kidney biopsy sample will be stored at the central laboratory in King’s College London for future analysis for different tests that will always be related to research. Some of the material may be sent to partner laboratories for specific tests related to this research.

If you decide to be part of the study you might need to spend 15 minutes longer in a small number of the outpatient clinic visits after the transplant so we can obtain various blood or urine samples. In addition you might be asked you to help us with the study by having an optional biopsy taken of the transplant kidney. This is an uncomfortable procedure and will involve you spending a day in hospital. If you decide not to have a biopsy taken, you can still be part of the study.

**WHAT DO I HAVE TO DO?**

If you decide to be part of the trial, we would ask you to sign the consent form on admission for the transplant. After the transplant you will then be looked after on the ward and in the outpatient clinic in the standard way. Following a transplant you will normally be expected to be an inpatient for about a week. If you are in the trial you will be asked to provide the extra blood and urine samples on the days when we remind you to do so.

**WHAT IS THE DRUG BEING TESTED?**

The drug being tested is called Mirococept. This is a new class of drug that can stick to the donor kidney and prevent inflammation. Part of the drug blocks a protein in your body called “complement”. This “complement” protein can damage a transplant kidney when it does not have a blood supply. Mirococept has been specially designed so that it is given to the kidney and not directly into the participant. The protein used to build the new drug is based on a natural protective human protein that has been reproduced and modified outside the body.

**ARE THERE ALTERNATIVES FOR TREATMENT?**

There are currently no licensed medications directed at resolving this particular issue that have been shown to effectively reduce the damage to a kidney when it has no blood supply.

**WHAT ARE THE POSSIBLE SIDE EFFECTS OF TAKING PART?**

There are unlikely to be any side effects from the drug that you will experience from taking part in this study. This is because the drug is given to the donor kidney after treatment of the kidney, and very little of the drug should enter your own circulation. This is known from previous evaluation of the drug in a small number of participants, when the drug is given into the donor kidney. However, you should be aware that any new medication can cause unexpected and serious side effects, even if these are rare. We have not experienced any such side effects in the small numbers of patients who have been treated so far, whether the drug has been given into the donor organ or, for other reasons, directly into the subject’s circulation.

**WHAT ARE THE POSSIBLE DISADVANTAGES AND RISKS OF TAKING PART?**

You must be aware that there is a risk to you if you agree to have a kidney biopsy as part of the trial. Following a planned transplant biopsy there is a risk that you might have some pain. Between 2 and 5% of participant s after a biopsy might have some bleeding. This normally stops of its own accord. However, a small number of this group might need a special X-Ray or surgery to stop the bleeding. Kidney biopsy is a routine test in some hospitals and in other hospitals is only done if the kidney transplant does not work properly. Your routine visits to the outpatient clinic may take a little longer than usual, due to the additional amount of blood and urine being taken for the research. This should add on no more than 15 minutes or so to each visit.

**EXPENSES AND PAYMENTS**

As the majority of the visits for this study are part of routine care, no travel expenses will be reimbursed. However, participants consenting to additional biopsies will be reimbursed reasonable travel expenses for this additional study visit.

HARM TO THE UNBORN CHILD

**For Women:**

There is no evidence that Mirococept could damage an unborn child, but this has not been tested. To be included in the study you will therefore need to have a negative pregnancy test on admission into hospital for the transplant. We do know that some of the routine transplant drugs can damage an unborn child and it is therefore common practice to require a negative pregnancy test at the time of transplantation.

Although there is no evidence that Mirococept will cause any foetal abnormalities we require that contraception is used for at least a month following the transplant.

**For Men:**

There is no evidence that Mirococept could damage sperm and consequently the foetus. However we require that contraception is used for at least a month following the transplant.

**WHAT ARE THE POSSIBLE BENEFITS OF TAKING PART?**

The purpose of the study is to see if we can reduce the chance of needing dialysis after the transplant. By reducing the damage to a kidney during the time it does not have a blood supply we also aim to find out if we can reduce the chance of long-term damage to the kidney. In general, a kidney transplant lasts on average 10 years and one of the aims is to see if the treatment will lengthen the lifespan of new kidney transplants. Since there is an equal chance that you would receive either the study medication or the standard treatment by participating in this study, there may be no direct benefit to you, even if the treatment is shown to be effective at the end of the entire study. However, your participation in the study may benefit future kidney transplant recipients.

**WHAT HAPPENS WHEN THE RESEARCH STUDY STOPS?**

The study team will follow your progress for one year after the transplant. We might contact you again after this time for longer-term follow up of the research.

**WHAT IF THERE IS A PROBLEM?**

An independent team of professionals are in place to monitor the safety of the study. If there are any problems with the study you will be contacted by the study doctors.

In the event that something goes wrong and you are harmed during this study there are no special compensation arrangements. However, the hospital continues to have a duty of care to you, whether or not you are participating in the study. Negligence of NHS staff will be indemnified by the NHS or professional indemnity schemes. If you are harmed due to someone’s negligence, then you may have grounds for a legal action but you may have to pay for your legal costs.

If you wish to complain, or have any concerns about any aspect of the way you have been approached or treated during the course of this study, the normal National Health Service complaints mechanisms should be available to you. Participation in this study does not affect your normal rights to complain about any aspect of your treatment and care (contact number details can be obtained from your hospital).

**WILL MY TAKING PART IN THIS STUDY BE KEPT CONFIDENTIAL?**

All information which is collected about you during the study will be kept strictly confidential. Any study information about you which leaves the hospital will have your name and address removed so that you cannot be recognized from it.

We have made sure that everyone involved in the study have laws and regulations that protect participant confidentiality (these regulations include the Data Protection Act 1998). Under this protection, only authorized representatives connected with the study are allowed access to the names of participants in the study. If we need someone else to access your details we will ask for permission from you by writing. Your hospital notes will however be reviewed by representatives of the sponsor and regulatory authorities.

**WHAT WILL HAPPEN TO THE RESULTS OF THE STUDY?**

The results of the study will probably be published within 4 years of the start of the study. None of the participants will be identified in the report. Once published the data is available to public access in specialized libraries.

A formal letter will automatically be sent to all participants informing them of the outcome of the study. You will be asked whether you wish to opt out from being contacted at the end of the study.

**WHO IS ORGANISING AND FUNDING THE STUDY**

The research is funded by a grant from the Medical Research Council (MRC). The study is being organised by doctors and scientists at the MRC Centre for Transplantation, King’s College London. This study is co-sponsored by King’s College London and Guy’s and St Thomas NHS Foundation Trust.

**WHO HAS REVIEWED THE STUDY?**

This study has been given a favourable ethical opinion for the conduct in the NHS by the NRES Committee London - South East.

**WHAT WILL HAPPEN IF I DON’T WANT TO CARRY ON IN THE STUDY?**

You can withdraw from the study at any time but information collected may still be used. Any stored blood or tissue samples that can still be identified as yours will be destroyed if you wish.

**INFORMING YOUR GENERAL PRACTITIONER**

If you agree, your GP will be informed that you are helping with the study. Other doctors not involved in the research, who may be treating you, may be informed of you being in the study.

**CONTACT FOR FURTHER INFORMATION**

**[Name of recruiting site PI]**

i.e. **Mr Martin Drage**

**[Name of research nurse]**

[Contact details including out of hours emergency 24 hour registrars’ office details]
