# Supplementary material for: A double-blind randomised controlled investigation into the efficacy of Mirococept (APT070) for preventing ischaemia reperfusion injury in the kidney allograft (EMPIRIKAL): study protocol for a randomised controlled trial
Source: Trials. 2017 Jun 6;18:255. doi: 10.1186/s13063-017-1972-x (PMC5461672; doi:10.1186/s13063-017-1972-x)
Supplement: Supplementary file 3 — Informed Consent Form. (DOC 57 kb) [file 13063_2017_1972_MOESM3_ESM.doc]

**
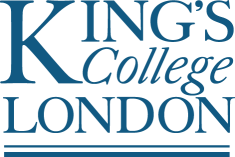
**

Centre Number:

Study Ref Number:

Patient Initials:

Patient Identification Number for this trial:

**CONSENT FORM**

Title of Project: **“EMPIRIKAL: Study on Kidney Transplantation”**

Name of Researcher:

**Please initial box**

1. I confirm that I have read and understand the information sheet dated

Version 5.1 22^nd^ October 2015 for the above study and have had the opportunity

to ask questions and discuss the study.

1. I have received enough information about the study and I have received satisfactory

answers to all the questions.

1. I understand that my participation is voluntary and that I am free to withdraw at any time,

without giving any reason, without my medical care or legal rights being affected.

1. I understand that sections of any of my medical notes may be looked at by responsible

individuals from my local NHS Trust or from Regulatory Authorities/sponsor

representatives where it is relevant to my taking part in research. I give permission for these

individuals to have access to my records.

1. I agree to the distribution of samples from my blood, urine and if taken a kidney

biopsy for future research studies.

1. **I wish / do not wish** to be contacted again should there be any longer-term follow up

of the research.

1. I agree for my GP to be informed of my participation on this study.

1. I agree to take part in the above study.

Name of Patient Date Signature (Patient)

Name of Person taking consent Date Signature
