# Supplementary material for: A double-blind randomised controlled investigation into the efficacy of Mirococept (APT070) for preventing ischaemia reperfusion injury in the kidney allograft (EMPIRIKAL): study protocol for a randomised controlled trial
Source: Trials. 2017 Jun 6;18:255. doi: 10.1186/s13063-017-1972-x (PMC5461672; doi:10.1186/s13063-017-1972-x)
Supplement: Supplementary file 4 — EMPIRIKAL Lab Manual. (PDF 1263 kb) [file 13063_2017_1972_MOESM4_ESM.pdf]

EMPIRIKAL TRIAL

CLINICAL TRIAL LABORATORY MANUAL

Version 4.0, dated 11th May 2016

Table of Contents

Approvals 3
Contact details 4
Abbreviations 5
Flow Diagram 1: Summary of EMPIRIKAL Sample Collections 7
Table 1: Research Samples Summary Table 8
1. Introduction 10
1.1 Funded Costs 10
1.2 Patient Packs 10
1.3 EMPIRIKAL Progeny Database for Sample Tracking 11
1.4 Equipment Requirements 12
2. Blood Sample Collection 12
2.1 Serum for complement and antibodies to Mirococept 12
2.3 Whole Blood Biomarker RNA (*cohort one patients only)* 14
2.4 Plasma sample for SDMA levels 15
3. Urine Sample Collection 16
3.1 Urine Filter Paper Samples 16
3.2 Urine Sample for C3a levels 18
3.3 Biomarker RNA urine *Guy's cohort one only* 18
4. Research Biopsy Tissue Collections 19
4.1 Guy's only Research Biopsy tissue collection 19
5. Shipping of samples 19
Appendix A: Barcode labelling 20
Appendix B: Research Samples Summary Table 21
Appendix C: Previous Versions 22

Approvals

A Double Blind Randomised Controlled Investigation into the efficacy of Mirococept (APT070) for preventing ischaemia-reperfusion injury in the kidney allograft (EMPIRIKAL)

Document Author: Laura Nichols, King's College London

The final version of the laboratory manual has been approved by:

Dr. Richard Smith, King's College London – Director of Protein Therapeutics

Signature ____ Date _______________

Contact details

ALWAYS SEND EMAIL NOTIFICATION TO **laura.nichols@kcl.ac.uk**
PRIOR TO THE SHIPMENT OF ANY SAMPLES

EMPIRIKAL Trial Office, Guy's Hospital

All EMPIRIKAL trial enquiries should be directed to:

Laura Nichols, EMPIRIKAL Clinical Project Manager, Division of Transplantation Immunology & Mucosal Biology, MRC Centre for Transplantation, King's College London, 5th Floor Tower Wing, Guy's Hospital, Great Maze Pond, London, SE1 9RT.
Email: laura.nichols@kcl.ac.uk
Tel: 020 7188 0616,
Fax: 020 7188 5660

Protein Therapeutics Laboratory, Guy's Hospital

Dr. Richard Smith/Dr. Roseanna Greenlaw, Division of Transplantation Immunology & Mucosal Biology, MRC Centre for Transplantation, King's College London, 5th Floor Tower Wing, Guy's Hospital, Great Maze Pond, London, SE1 9RT.
Email: richard.a.smith@kcl.ac.uk; roseanna.greenlaw@kcl.ac.uk
Tel: 0207188 8567/1527
Fax: 020 7188 5660

WellChild Laboratories

Professor Neil Dalton, WellChild Laboratory. King's College London, Arctic (1st Floor), Evelina Children's Hospital, St Thomas' Hospital, Lambeth Palace Road, London, SE1 7EH.
Tel: 0207 188 0159

Abbreviations

Abbreviation	Definition
Abs	Antibodies
AE	Adverse Event
ALT	Alanine aminotransferase
APT070	Mirococept
AR	Adverse Reaction
AUC	Area Under Curve
BMI	Body Mass Index
BPAR	Biopsy-proven acute rejection
CCD	Cumulative Cohort Design
CCP	Complement Control Protein
CMV	Cytomegalovirus
CNI	Calcineurin inhibitor
CRF	Case Report Form
DBD	Donation after brain-death
DCD	Donation after Circulatory Death
DGF	Delayed Graft Function
DSMB	Data Safety Monitoring Board
eGFR	Estimated Glomerular Filtration Rate
GCP	Good Clinical Practice
αGST	Alpha glutathione S-transferase
GGT	Gamma-glutamyl transferase
HBV	Hepatitis B Virus
HCV	Hepatitis C Virus
HIV	Human immunodeficiency virus
HLA	human leukocyte antigen
HTAct	Human Tissue Act
IMP	Investigational Medicinal Product
IRI	Ischaemia Reperfusion Injury
KHP- CTO	King's Health Partners Clinical Trials Office
MDRD	Modification of diet in renal disease study equation
MAC	Membrane attack complex
MED	Minimum Effective Dose
MHRA	Medicines & Healthcare products Regulatory Agency
MMF	Mycophenolate mofetil
MRC	Medical Research Council
MRIS	Medical Research Information Service
NICE	National Institute for Clinical Excellence

NGAL	Neutrophil gelatinase-associated lipocalin
NAG	N-acetyl-β-D-glucosaminidase
NRES	National Research Ethics Service
PBS	Phosphate buffered saline
PJP	Pneumocystis jiroveci pneumonia
PK	Pharmacokinetics
PTL	Protein Therapeutics Laboratory
PTLD	Post-transplant lymphoproliferative disorder
RCA	Regulators of complement activation
REC	Research Ethics Committee
RBP	Retinol binding protein
RNA	Ribonucleic acid
RT	Room temperature
SAE	Serious Adverse Event
SCr	Serum creatinine
SDMA	Symmetric dimethylarginine
SOP	Standard Operating Procedure
SPC / SmPC	Summary of Product Characteristics
SST	Serum Separating Tube
SUSAR	Suspected Unexpected Serious Adverse Reaction
T_0	Time zero- before transplantation of the perfused organ
T_1	After transplant 1 hour post perfusion with blood
T_4	After transplant 4 hours post perfusion with blood
TMG	Trial Management Group
TSC	Trial Steering Committee
UW	University of Wisconsin solution
Vz	Mean volume of distribution
WCL	WellChild Laboratory

Flow Diagram 1: Summary of EMPIRIKAL Sample Collections

Does your Centre have an HTA research license and access to a refrigerated centrifuge with swing-out bucket rotor (spinning down) facilities?

- No — *Note: Your centre will require a HTA license to participate in this trial.
- Yes

Does your Centre have -80° and -20° C freezer storage facilities?

- Yes
- No — *Note: Your centre will require these facilities for the storage of samples.

Yes → Sample Processing and Storage Guidance

Blood Processing

Serum Samples
Collect blood into an orange top tube. Mix by inverting five times and allow to clot for 30 minutes at room temperature. After clotting, centrifuge at 1200g for 10 minutes. Collect aliquots (2x minimum ½ filled cryovials) for serum Mirococept, serum antibodies to Mirococept and serum complement levels *depending on time point*

Plasma Samples for SDMA
Collect blood into a green top tube. Mix by inverting eight times and centrifuge at 1500g for 10 minutes at 4°C as soon as possible (max. within 4 hours). Collect an aliquot for SDMA (1x cryovial) *keep cold*

Whole Blood Samples (Plasma RNA)
Collect whole blood into Tempus Blood RNA tubes (fill 2 tubes per time point). Mix gently eight times. No centrifugation is needed.

Urine Processing for –80°C Storage

For all urine samples collect up to 100ml of urine into a Sarstedt pot. **(KEEP COLD)**

Urine Filter Papers
Prior to urine centrifugation spot urine on to all dots on a filter paper.

C3a
Collect 10-50ml from original Sarstedt pot. Centrifuge at 1500g for 4°C for 15 minutes. Collect supernatant (1 x 1.5ml) *keep cold and process as soon as possible*

***Guys' cohort one only* RNA samples**
If there is urine remaining keep the original Sarstedt pot in the fridge or on ice and contact Mano (Manohursingh.runglall@kcl.ac.uk)

– 80°C Blood Storage
Serum
•Mirococept
•Antibodies to Mirococept
•Complement
Plasma
•SDMA

-20°C Blood storage
• RNA Whole blood tempus tubes

– 80°C Urine Storage
•C3a
• Urine RNA

Room Temperature
•Urine Filter Paper

All centrifugation needs to use a swing-out bucket rotor
Centrifugation speeds are given as 'g' not rpm

Table 1: Research Samples Summary Table

Assessment	BL/ Day 0	Day 1	Day 2	Day 3	Day 4	Day 7	Wk 2	Wk 3	Wk 4	Wk 5	Wk 6	Wk 7	Wk 8	Wk 9	Wk 10	Wk 11	Wk 12	Wk 24	Wk 36	Wk 52
**Visit Window**	**Completed on day**					**+/- 2 days**	**Anytime in specified week (7 day window)**											**+/- 2 weeks**		
**Serum for complement levels**	x	Δ	Δ	Δ	Δ		Δ		Δ											
**Serum for antibodies to Mirococept**							Δ		Δ						Δ					
**Plasma SDMA levels**	Δ	Δ	Δ			Δ														Δ
**Urine filter papers**		Δ	Δ			Δ														Δ
**Urine for C3a levels**		Δ	Δ					Δ							Δ	Δ	Δ	Δ	Δ	Δ
**Whole blood RNA**		Δb	Δb			Δb	Δb		Δb				Δb				Δb	Δb	Δb	Δb
**Guy's Patients Only**																				
**Biopsy**	Δa																Δ			
**Serum for complement levels**	Xc																			
**Serum for Mirococept levels**		Δ																		
**Urine RNA**		Δb	Δb			Δb	Δb		Δb				Δb				Δb	Δb	Δb	Δb

Key

Symbol	
• **BL** = baseline	• **Δb =** cohort 1 patients only
• **X** = essential	• **Δ** = desirable
**Assessment Windows**	
Assessments must be completed on day specified	
Assessments completed **+/- 2 days** of this time point	
Assessments completed **anytime in that 7 day** window	
Assessments completed **+/- 2 weeks** of that time point	
**Guy's Patients Only**	
• **Δa = ½ of 1** core taken before organ perfusion & 1 post perfusion with blood	• **Δc =** to be taken T_0 (before transplantation), 1hr and 4hr following perfusion with blood)

NB
The above assessments all form part of the routine management of renal transplant assessments with the exception of the following;

- serum for complement levels
- serum for antibodies to Mirococept
- serum for Mirococept levels (only at Guy's site)
- plasma for SDMA levels
- urine for markers of tubular damage (RBP, NAG and NGAL)
- urine RNA
- urine for C3a levels
- whole blood RNA
- renal biopsy for research (only collected at Guy's site)

- **Where an "x" is contained within a field this denotes that the associated data is essential and if missed will be classified as a protocol deviation.**
- **Where an "Δ" is contained within a field this donotes that the associated data is desirable and while every effort should be made to record these data missing data will NOT constitute a protocol deviation.**

1. Introduction

The purpose of this manual is to describe the collection, site processing and transportation of blood, urine and biopsy tissue samples for the EMPIRIKAL trial. This document should be used in conjunction with the EMPIRIKAL trial protocol.

The following research samples are required, in addition to the routine laboratory investigations outlined in the protocol, from **all patients at all participating centres** unless otherwise stated:

1. Serum for complement levels
2. Serum for antibodies to Mirococept
3. Plasma for SDMA levels
4. Urine for markers of tubular damage – RBP, NAG, NGAL albumin and creatinine (on filter papers)
5. Urine for C3a levels
6. ***Cohort one only*** Whole blood RNA for biomarkers

<u>Guy's site only</u>

1. Serum for Mirococept levels
2. ***Cohort one only*** Urine for biomarkers
3. Renal biopsy specimens for research (optional)

Although serum, plasma and spun urine do not fall under the Human Tissue Act (HTAct) as relevant material, all samples will be processed, tracked and stored according to HTAct 2004 and the EU Directive Guidelines.

All participants' samples will be shipped, when requested, from recruiting centres to the Protein Therapeutics Laboratory (PTL). The PTL will sort and send relevant samples to the WellChild Laboratory (WCL).

In order to preserve the integrity of the patient samples (and comply with the clinical trials regulations), we request that staff at participating centres collect, handle, process and store patient samples at their centre in accordance with these instructions.

1.1 Funded Costs

Sample collection for the study has been funded by the Medical Research Council (MRC) via the study grant. The trial team will supply all of the consumables associated with sample collection and storage. Collection and transportation, on dry ice, to the PTL will be arranged and paid for by the EMPIRIKAL Clinical Project Manager.

1.2 Patient Packs

Each participating centre will receive EMPIRIKAL patient packs for the collection of non-routine samples required for the trial. Packs will contain:

- Serum vacutainer tubes (Orange topped tubes)

- Lithium heparin vacutainer tubes (Green topped tubes)
- Tempus blood RNA tubes (Blue topped tubes)
- 100ml Sarstedt urine pots
- Stickers to be put onto urine pots
- 1.5ml cryovials
- Urine filter cards in foil bags with dessicant tablet
- Sterile pipettes
- 50ml Falcon tubes
- Pre-paid mailing envelopes for the return of filter papers (address labels also provided)
- Stickers for labelling blood vacutainer and tempus tubes
- Copies of Baseline Sample Tracking Forms
- Copies of Post Transplant Sample Tracking Forms
- Labelled box for storage of cryovials at -80°C
- Labelled Tempus tube rack for storage of tempus tubes at -20°C
- Blank paper cryovial box map (to document storage location of samples at -80°C)
- Blank paper tempus rack map (to document storage location of samples at -20°C)
- Indivudual sample barcodes to affix to cryovials once filled (see Appendix A)

Guy's only

- 1.5ml cryovial containing 1.5ml Michels solution (will be stored in the designated theatre fridge until used, thereafter at room temperature).

The above supplies will be sent to site initially and should you run low on anything please let the EMPIRIKAL Clinical Project Manager know and further supplies will be sent.

Centres will be provided with storage boxes and racks for the storage of samples at their site. These boxes and racks are to be used when shipping samples back to the PTL and replaced with new boxes and racks.

1.3 EMPIRIKAL Progeny Database for Sample Tracking

All EMPIRIKAL participants' samples must be logged on the EMPIRIKAL Progeny database, unless stated otherwise in the instructions below. Participants at each centre will be pre-generated on the database; numbered 001, 002, and 003 at each site and so on. The number will match that given to the patient at randomisation on MACRO and Sealed Envelope (refer to the protocol and randomization step by step instructions for further information). One Progeny log in will be provided per centre.

Following the randomisation of the participant, a Progeny trained member of staff will need to log in to the Progeny system https://labsamples.kcl.ac.uk/#1 and enter basic participant's details. Please refer to the EMPIRIKAL Progeny Database User Guide for further detail on using the Progeny system.

In addition to Progeny, paper records of samples taken will be kept; recording the time and date a sample was collected and processed. Research Sample Tracking Forms will be provided to site (two forms; one to be used for baseline samples only and another to be used at all time points for all samples collected post-transplant).

1.4 Equipment Requirements

The following equipment is essential and required to complete sample processing and/or storage:

- -80oC freezer for storage of sera, plasma and urine C3a samples.
- -20oC freezer for storage of whole blood Tempus tubes.
- Refrigerated swing-out bucket centrifuge for spinning samples.

All participating centres confirmed they had access to the above facilities prior to being approved as a research site.

2. Blood Sample Collection

Key points to note for blood sample collection:

- **Samples for serum (complement, antibodies for Mirococept and Mirococept) should be collected in the orange topped tubes and, after mixing well, can be left unprocessed for up to 72 hours following collection. This means samples can be collected over the weekend and left at room temperature prior to processing the next working day.**

- **Samples for plasma (SDMA) should be collected in green topped tubes and must be processed within 4 hours of collection. If this isn't going to be possible (e.g. over the weekend) do not collect the sample.**

- **Whole Blood (RNA) should be collected in blue topped tempus tubes and after mixing well can be left for up to 72 hours before being stored at -20oC. This means samples can be collected over the weekend and left at room temperature prior to storage the next working day.**

- **All blood samples must be stored at room temperature following collection prior to processing.**

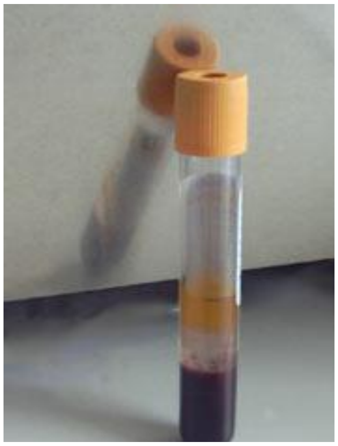

Figure 1: Orange topped serum separator tube

2.1 Serum for complement and antibodies to Mirococept

Use the method below for processing both the serum complement samples and serum antibodies to Mirococept samples. At Guy's only this method should also be used when processing the serum Mirococept samples.

1. Fill the orange topped tube to the fill line and invert 5 times to mix then leave to clot for 30 minutes at room temperature (Figure 1).

2. Record the date and time of sample collection on the research sample tracking form.

3. Centrifuge the tube at **room temperature** at **1200g** for **10 minutes** in a swing-out bucket rotor to obtain serum (Figure 3).

Unspun orange top blood tube

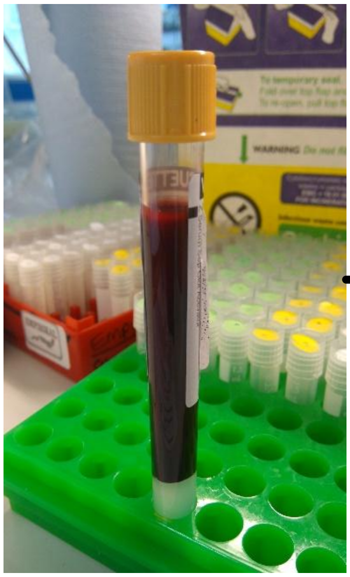

Centrifuge at 1200g for 10 mins at room 20°C

Orange top blood tube after centrifugation

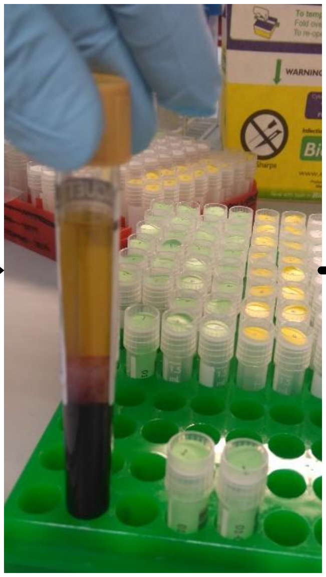

Collect serum

4. While the sample is being spun record the date and time of sample processing on the research sample tracking form.

5. Aliquot serum (2 samples of roughly 0.5ml) into the supplied cryovials (Figure 2). Affix correct Progeny barcode (pre-provided to site) onto the outside of the cryovial. (For serum complement levels these barcodes are white. For serum antibodies to Mirococept and serum Mircococept levels these barcodes are green.) Take the circular white or green sticker (depending on the serum sample type, and stick on the top of the cryovial lid (see Figure 4).

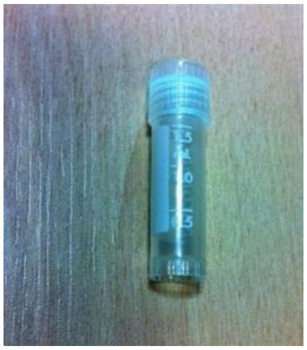

Figure 2: cryovial

6. Put both aliquots in the next free slots in the provided cryovial box, making a note of their location on the paper cryovial box map.

7. Store at -80oC.

8. Using the data on the sample tracking forms and cryovial box map record the date and time of sample collection, processing, storage box number and positon in the Progeny database.

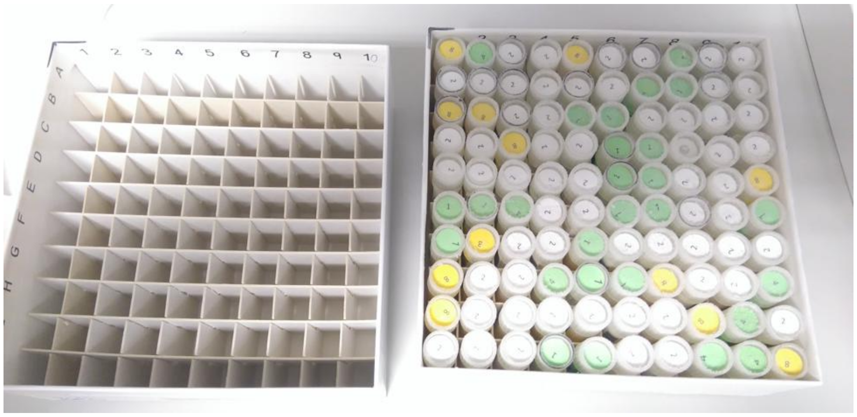

Figure 4: Labelled cryovials in box

2.3 Whole Blood Biomarker RNA (*cohort one patients only)*

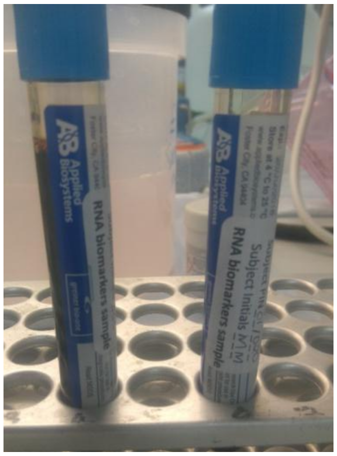

Figure 4: Blue topped tempus tube

1. At each time point fill two blue topped tempus tubes to the fill line. (see Figure 4).

2. Immediately mix by gently inverting the tube eight times.

3. Label the tube with the patient PIN (PXXXXX) and timepoint, use labels provided.

4. Record the date and time of sample collection on the research sample tracking form.

5. Put both blue topped tubes in the next free slots in the provided tempus tube rack, making a note of their location on the paper tempus tube rack map (see Figure 5).

6. Store at -20oC and record the time the samples were frozen on the research sample tracking form.

7. Log the location of the blue topped tubes on Progeny (rack number and poisiton only). There are no barcodes for these samples, just write the patient PIN and timepoint on the tubes.

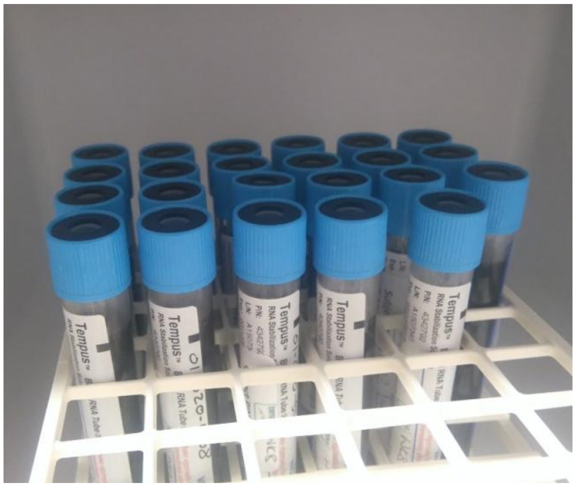

Figure 5: Tempus tubes stored in tempus rack

2.4 Plasma sample for SDMA levels

N.B. This sample needs to be processed within 4 hours of collection; if processing isn't possible within 4 hours of collection (for example sample taken out of hours or at weekends) there is no need to collect the sample

1. Fill the green topped tube to the fill line and invert 5 times to mix.

2. Record the date and time of sample collection on the research sample tracking form.

3. Centrifuge the tube at **1500g** for **10 minutes** at **4oC**.

4. While the sample is being spun record the date and time of sample processing on the research sample tracking form.

5. Aliquot plasma (1 sample of about 1.5ml) into the supplied cryovial. Affix correct Progeny barcode (pre-provided to site) to the outside of the cyrovial. (For plasma SDMA level the barcode is yellow). Take the circular yellow sticker with a “8” and stick on the cryovial lid (see Figure 6).

6. Put the aliquot in the next free slot in the provided cryovial box, making a note of it’s location on the paper cryovial box map.

7. Store at -80oC.

8. Using the data on the sample tracking forms and cryovial box map record the date and time of sample collection, processing, storage box number and positon in the Progeny database.

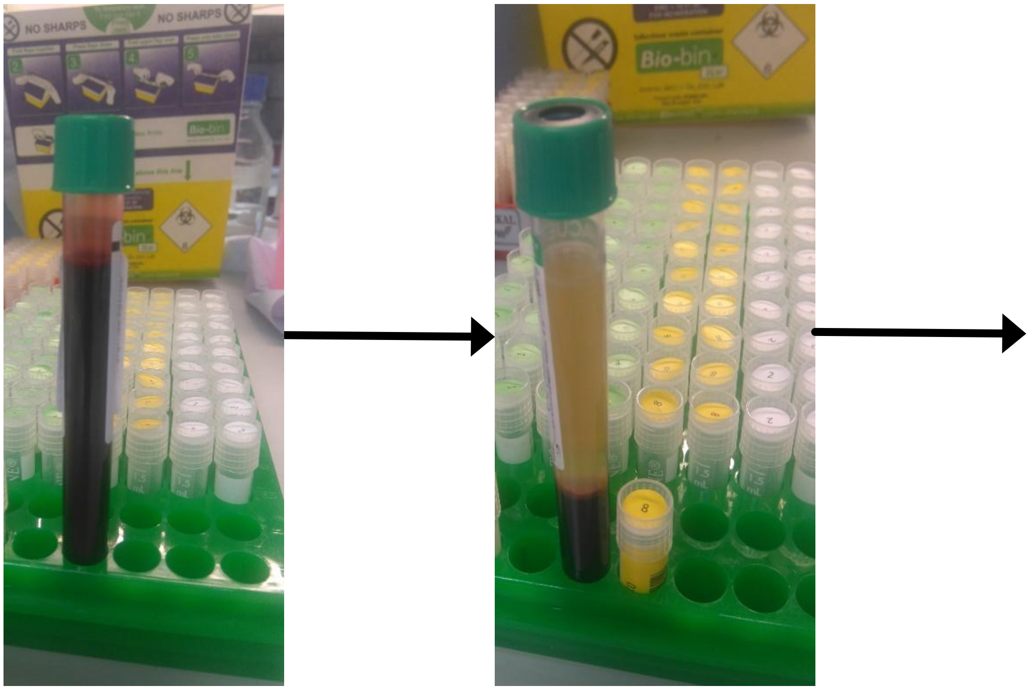

Take plasma

3. Urine Sample Collection

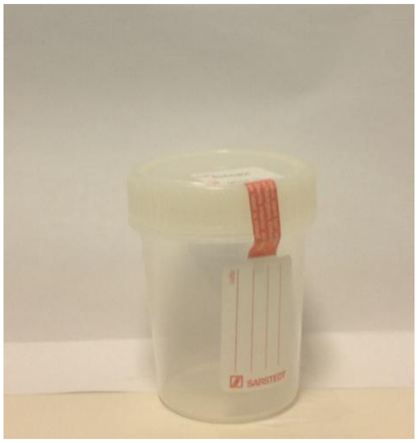

Figure 7: 100ml Sarstedt pot

Collect up to 100ml of urine in 100ml Sarstedt pot (Figure 7) at the appropriate time points, and complete the processing from that single sample as required by the assessment schedule:

- Urine for markers of tubular damage on filter papers
- Urine for C3a levels
- ***Guy's cohort 1 Only*** *Using any remaining unprocessed urine* **-** Urine Biomarker RNA

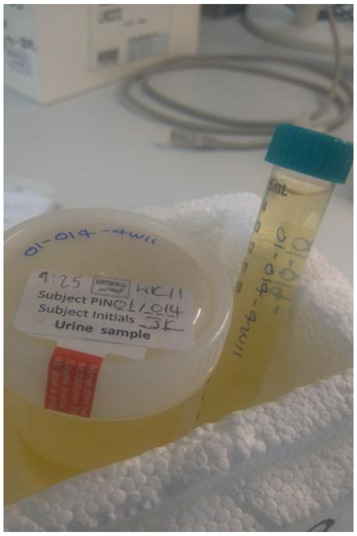

Figure 8: Urine samples on ice prior to processing

Important points to note for urine sample collection:

Use one urine sample for all processing of urine at any given time point.

Urine collected must be processed within 4 hours of collection. If this isn't going to be possible (e.g. over the weekend) do not collect the sample.

Urine must be kept cold between collection and processing so keep unprocessed urine in the fridge or on ice (Figure 8).

3.1 Urine Filter Paper Samples

1. Take one foil packet per time point, remove the filter paper from the packet.

2. Take a sterile pipette and using the sample collected in the Sarstedt pot spot urine onto each of the circles on the filter card provided.

3. Dry at room temperature for one hour. While the filter paper is drying record the date and time of sample processing on the research sample tracking form.

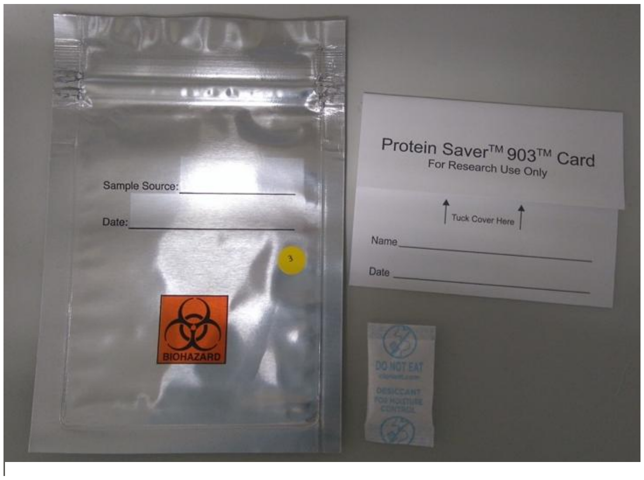

Figure 9: Urine filter paper, desiccant and bag

4. Affix one of the correct Progeny barcodes (pre-provided to site) onto the filter paper. (For urine filter paper samples these barcodes are yellow) and complete name and date sections on the filter card, for name use patient PIN, then place the card in the foil storage bags containing the desiccant tablet.

Front labelling of filter paper

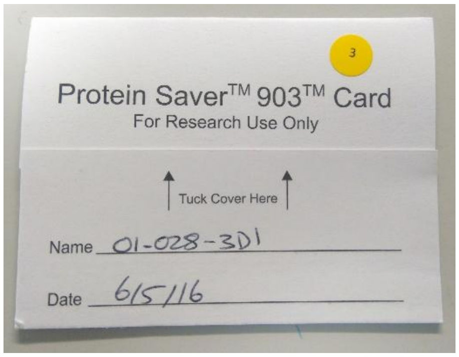

Back labelling of filter paper

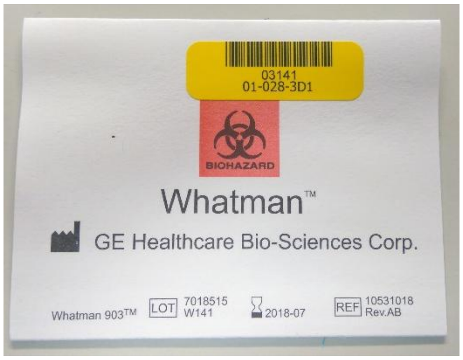

Labelled filter storage bag

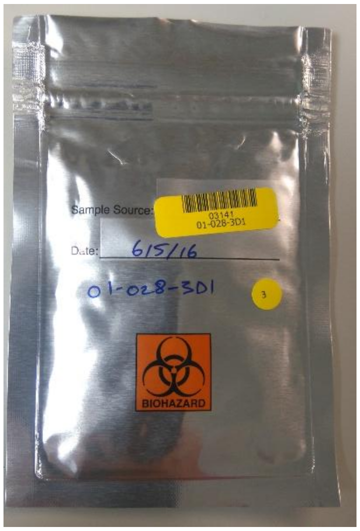

5. On the outside of the foil storage bags affix the second Progeny barcode relating to that sample (for urine filter paper samples two identical barcodes are provided per sample required) and in marker pen write the patient ID number and timepoint of sample. (For example "P08005 D2").

For each time-point, put a drop of unspun urine onto all spots on one filter paper

If possible, prepare urine filters on days 1, 2 and 7. Once dry, store in their envelope at room temperature

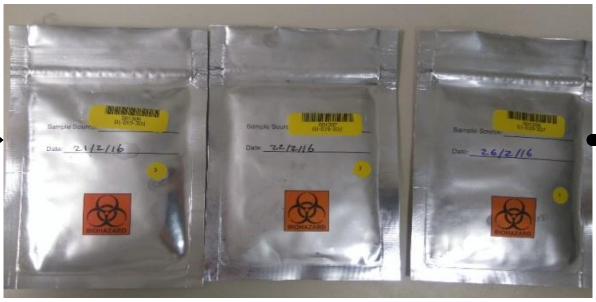

Use 1 envelope per subject to send filters to PTL - Maximum of 3 filters per envelope.

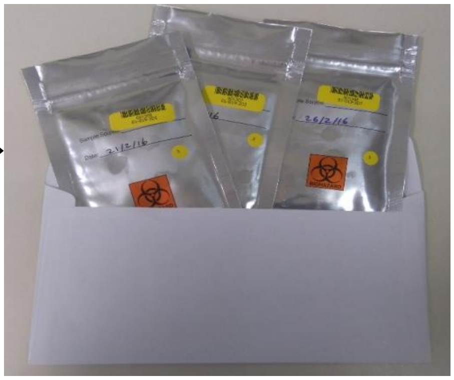

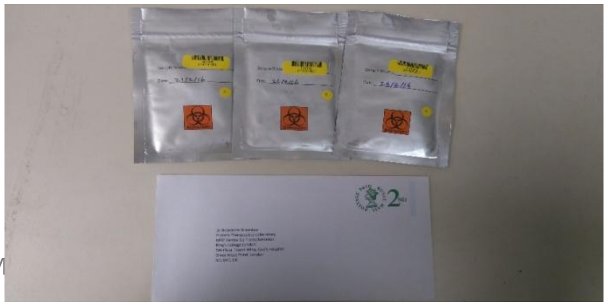

6. Samples collected for the **same** patient on **days 1, 2 and 7** should be batched together and all three mailed to the PTL (by day 12) in the same envelope using the prepaid envelopes and address labels provided to site. Use one envelope per patient – do not mix patient samples in the same envelope even if not all the timepoints were collected.

7. Send an email to roseanna.greenlaw@kcl.ac.uk and laura.nichols@kcl.ac.uk with date and which samples were sent. There is no need to log the urine filter paper samples on the Progeny database.

8. Repeat steps 1 to 7 for week 52 sample and mail to PTL within 5 days.

3.2 Urine Sample for C3a levels

N.B. if a urine filter card is required do this first then prepare the urine C3a samples as follows:

1. Put between 10 – 50ml urine into a suitable container and centrifuge at **1500g** at **4oC** for **15 minutes**. 50ml falcon tubes have been provided to sites for this but any tube can be used that best fits the centrifuge to be used.

2. While the sample is being spun record the date and time of sample processing on the research sample tracking form.

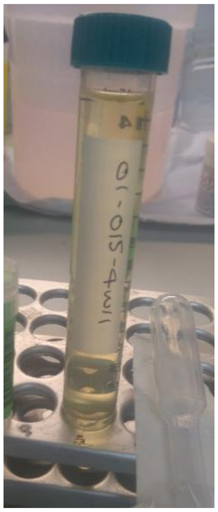

Figure 10: Urine in tube for spinning

3. Aliquot supernatent (1 sample of roughly 1.5ml) into the supplied cryovial. Affix correct Progeny barcode (pre-provided to site) onto the outside of the cryovial. (For urine C3a levels these barcodes are green.) Take the circular green sticker sticker with a “4” and stick on the cryovial lid.

4. Put the aliquot in the next free slot in the provided cryovial box, making on note of it’s location on the paper cryovial box map.

5. Store at -80oC.

6. Using the data on the sample tracking forms and cryovial box map record the date and time of sample collection, processing, storage box number and positon in the Progeny database.

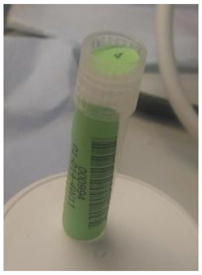

Figure 11: Supernatent in labelled cryovial

3.3 Biomarker RNA urine *Guy’s cohort one only*

1. If there is any remaining urine after completing all other urine sampling keep the remaining urine in the Sarstedt pot in the fridge, or on ice, and contact Mano (Manohursingh.runglall@kcl.ac.uk) as soon as possible.

4. Research Biopsy Tissue Collections

Trial biopsy collection for research purposes will initially be at the Guy's site only. The study may request additional support from other sites if additional month 3 biopsy samples are needed.

4.1 Guy's only Research Biopsy tissue collection

Important points to note for biopsy sample collection:

- **Trial biopsy samples will be collected and preserved in Michels solution. Michels solution must be handled with care and only use when wearing protective clothing, including gloves.**
- **Biopsy samples should be stored at room temperature and will be transferred to PTL within 72 hours +/- 2 days.**

1. At the time of the transplant two biopsy samples will be taken as per local practice; pre perfusion with blood and IMP (labelled A) and after transplantation following perfusion with blood and IMP (labelled B). Standard clinical care at Guy's hospital is to take two cores; it has been agreed that ½ of one of these cores can be given for trial purposes.

2. The designated ½ of one of the cores taken is to be immersed in a cryovial containing 1.5ml of Michels solution. (Prior to use the cryovials are stored in the Soltran fridge in theatres; for each patient two cryovials should be used one labelled "A" for the pre perfusion biopsy and one labelled "B" for the post perfusion biopsy.

3. The biopsy sample tracking form should be completed with the time both biopsies were taken. The samples should then be stored at room temperature until collection.

4. The sample will be processed (snap-frozen), within 5 days of being taken, by a member of the PTL trial team. The date and times of collection and processing will then be added to the Progeny database by the PTL trial team.

5. If the patient consents to the optional 3 month biopsy this should be processed as above.

5. Shipping of samples

The EMPIRIKAL Project Manager will arrange and pay for dry ice shipment of all stored samples to the PTL during the trial. With the exception of urine filter papers, which will be sent in the prepaid envelopes provided as outlined in section 3.1 above.

If your centre runs out of storage space please contact the EMPIRIKAL Clinical Project Manager to arrange sample collection.

Appendix A: Barcode labelling

The table below outlines the bar-coding system code.

**Time points**
T_0= baseline before transplantation with perfused organ
D = day
W= week
T_1 – 1 hour after transplantation (+ 1 hour)
T_4 – 4 hours after transplantation (+ 4 hours)

<table>
<tr><th>Samples</th><th>Vessel</th><th>Sample code</th><th>Barcode label color</th><th>Number of samples to be collected</th><th>Number of aliquots to be stored</th></tr>
<tr><td>Serum antibodies to Mirococept</td><td>Cryovial</td><td>1</td><td>Green</td><td>1</td><td>2</td></tr>
<tr><td>Serum Mirococept</td><td>Cryovial</td><td>1</td><td>Green</td><td>1</td><td>2</td></tr>
<tr><td>Serum Complement</td><td>Cryovial</td><td>2</td><td>White</td><td>1</td><td>2</td></tr>
<tr><td>Plasma SDMA</td><td>Cryovial</td><td>8</td><td>Yellow</td><td>1</td><td>1</td></tr>
<tr><td>Whole blood RNA</td><td>Tempus tube</td><td>N/A</td><td>N/A use standard Tempus label</td><td>2</td><td>N/A no processing required freeze tube directly</td></tr>
<tr><td>Urine Filter Papers</td><td>Filter paper</td><td>3</td><td>Yellow</td><td rowspan="3">1</td><td>1</td></tr>
<tr><td>Urine C3a</td><td>Cryovial</td><td>4</td><td>Green</td><td>1</td></tr>
<tr><td>Guy's Only - Urine RNA Biomarkers</td><td colspan="3">N/A</td><td></td></tr>
<tr><td>Guy's Only - Biopsy</td><td>Cryovial</td><td>7</td><td>Green</td><td>1</td><td>N/A</td></tr>
</table>

Patient Sample Labelling

The first two digits identify the site e.g. Guy's = 01 and the next three is the number of the patient at that site.

**01**	**001**	**2**	**W4**	**1**
Site	Patient PIN	Sample code	Time point	Aliquot(s)

On Progeny this sample would be displayed as 01-001-2W4_1. This means: Guy's Patient number 1 serum complement sample at week 4 and this is aliquot 1.

**P05**	**011**	**8**	**T0**
Site	Patient PIN	Sample code	Time point

On Progeny this sample would be displayed as 05-011-8T0. This means: St. George's patient number 11 plasma SDMA sample at baseline (time 0).

Appendix B: Research Samples Summary Table

Research Samples Summary Table							
**Sample**	**Time point Collected**	**Collection tube**	**Spin Speed/Time/ Temperature**	**Instructions**	**Aliquots**	**Barcode Colour/ Number**	**Storage**
Serum for complement levels	Day 0, 1, 2, 3, 4 weeks 2 and 4	**Orange** topped tube	**1200g** for **10mins** at **room temp**	Invert 5 times, allow to clot for 30 min. Centrifuge within 72 hrs.	2	White/2	-80°C
Serum for antibodies to Mirocecept	Weeks 2, 4 and 10	**Orange** topped tube	**1200g** for **10 mins** at **room temp**	Invert 5 times, allow to clot for 30 min at room temp. Centrifuge within 72 hrs.	2	Green/1	-80°C
Guy's Only - Serum Mirocecept levels	Day 1	**Orange** topped tube	**1200g** for **10 mins** at **room temp**	Invert 5 times, allow to clot for 30 min at room temp. Centrifuge within 72 hrs.	2	Green/1	-80°C
Plasma for SDMA levels	Day 0, 1, 2, 7 week 52	**Green** topped tube	1500g for 10 min at 4 °C	Invert 8 times, process immediately but at least within 4 hours.	1	Yellow/ 8	-80°C
Urine for C3a levels	Day 1, 2, week 3, 10, 11, 12, 24, 36 and 52	Urine pot transfer 10ml to falcon tube	1500g for 15 mins at 4 °C	Keep sample cold after collection and during processing. Process within 4 hours.	1	Green/4	-80°C
Urine filter papers	Day 1, 2, 7 week 52	Urine pot use pipette to spot urine on to each spot on filter card	Not Applicable	Use pipette to spot urine on to each spot on filter card. Leave to dry for at least 1 hour. Place dry card in silver envelope, Write on outside of silver envelope patient ID and time point of sample	N/A	Yellow/ 3	Mail within 5 days batching together day 1,2 and 7 – using prepaid envelope and address label provided
Whole Blood for RNA	Day 1, 2, 7, week 2, 4, 8, 12, 24, 36 and 52	**Blue** topped Tempus tube	Not Applicable	Fill two tubes at each time point. Invert tubes 8 times	N/A	N/A	-20°C

Appendix C: Previous Versions

Revision of Version / Date/ Reason for Amendment	Superseded by Version and date
Version 1, dated 16th December 2013: administrational updates to centrifuging methods.	Version 2, dated 15 April 2015
Version 2, dated 15th April 2015: updated due to protocol amendment changing the sample collection schedule; changes to wording/formatting for clarity	Version 3, dated 20th July 2015
Version 3, dated 29th April 2016: updated with simplified instructions and clarity for sites	Version 4, dated 29th April 2016
