# Supplementary material for: A double-blind randomised controlled investigation into the efficacy of Mirococept (APT070) for preventing ischaemia reperfusion injury in the kidney allograft (EMPIRIKAL): study protocol for a randomised controlled trial
Source: Trials. 2017 Jun 6;18:255. doi: 10.1186/s13063-017-1972-x (PMC5461672; doi:10.1186/s13063-017-1972-x)
Supplement: Supplementary file 5 — Simulation study. (DOC 231 kb) [file 13063_2017_1972_MOESM5_ESM.doc]

**Simulation Study**

The choice of rules to decide the dose-allocation of the next cohorts is vital in the performance of an adaptive design. In order to find the most efficient rules for our study we have used the Compass software (www.cytel.com) to perform **trial simulations** under different scenarios. The conditions assumed in the simulation study were these:

- Total sample size of 560 transplants
- 35% of DGF in the placebo group
- A reduction of 10% compared to placebo is the minimum clinically significant effect.
- Target dose is between 10 and 15 mg (where the proportion of patients free of DGF is approximately 0.75).
- First cohort is randomized to placebo or 10mg dose
- Allocation ratio (Pbo:Drug) = 1:2 (27 placebo/53 Drug per cohort)

We simulated 200 trials under each of 4 different scenarios of dose response (Figure 2) in order to choose allocation rules that would give us the right answer in most cases:

1. A null distribution - no effect.
2. A quadratic curve, with monotonic growth
3. A non-monotonic curve where performance decreases with doses higher than 15 mg.
4. A four parameter logistic function


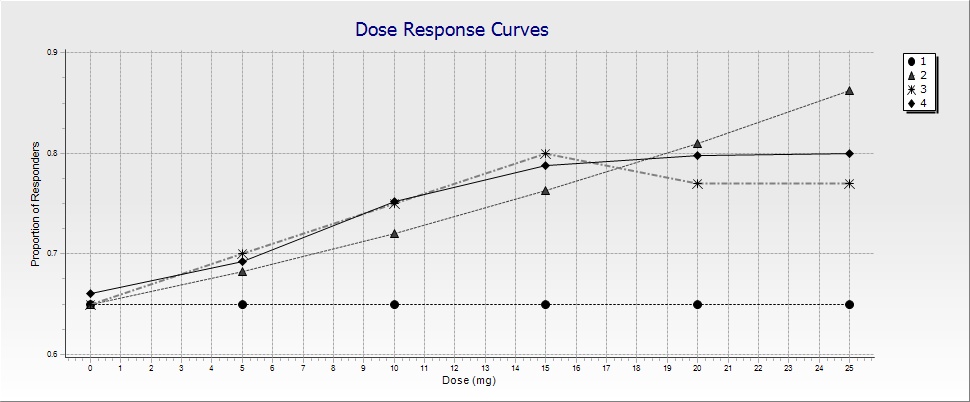


Figure 2. Dose response curves simulated

The results showed that the following allocation rules provided optimal performance:

- *If t-statistic < -0.01* 🡪 *Increase one dose (e.g. from 10mg to 15mg)*

- *If -0.01 < t-statistic < 1.5* 🡪 *Repeat the current dose (e.g. keep 10mg)*

- *If t-statistic >1.5* 🡪 *Decrease one dose (e.g. from 10mg to 5mg)*

The t-statistic for binary categorical outcomes uses a standard normal approximation of the binomial :  (p1-p2**-C1**)/sqrt( (p1q1/n1) + (p2q2/n2) ), where p1 and p2 are the *estimated* responses at the current dose for treatment and placebo groups, and q1=1-p1 and q2=1-p2; C1 is the target minimum clinically significant difference, i.e. 0.10 in this trial. A t-statistic = 0 implies a difference in the rate of DGF between the drug and placebo groups of 0.10, the minimum clinically significant; a positive t-statistic implies that the difference in DGF rate between placebo and drug is larger than 0.10, and a negative t-statistic implies that the difference is lower than 0.10.


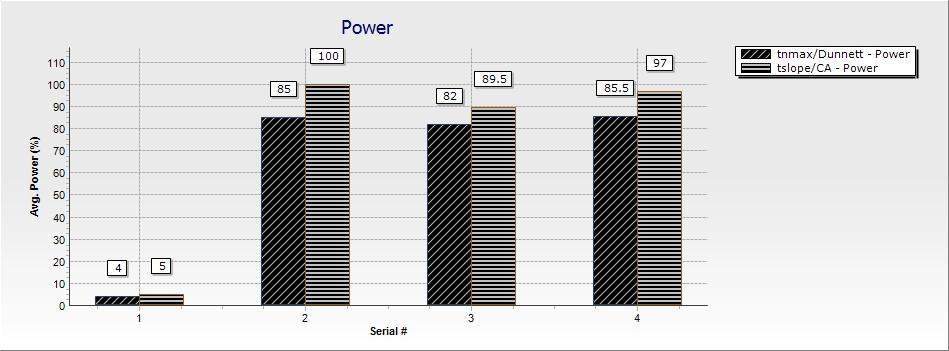


Figure 3: Statistical Power according to simulation results

Figure 3 shows the power to detect differences in response between placebo and drug at the dose given to the largest number of subjects (tnmax/diagonal stripped), and the power to estimate the dose-response curve (tslope/horizontal stripped) for each of the 4 dose-response scenarios.

Power reached .80 for the 3 dose-response scenarios, but was slightly lower for the non-monotonic response curve (serial #3). Under the absence of effect (serial# 1), only 5% of simulated trials found a significant effect, consistent with a 5% type I error. Other measures of effectiveness were also satisfactory; i.e. an effective dose was found in 87%, 64% and 90% of the simulated trials for the curves 2, 3 and 4 respectively. The percentage of simulations in which the target dose or the nearest one was selected were 93%, 79% and 87%.

Note that, when there is an effect, most patients were effectively allocated around the target dose (i.e. the minimum effective dose, which results in a reduction of ~10% in DGF between placebo and drug).
