# Supplementary material for: A double-blind randomised controlled investigation into the efficacy of Mirococept (APT070) for preventing ischaemia reperfusion injury in the kidney allograft (EMPIRIKAL): study protocol for a randomised controlled trial
Source: Trials. 2017 Jun 6;18:255. doi: 10.1186/s13063-017-1972-x (PMC5461672; doi:10.1186/s13063-017-1972-x)
Supplement: Supplementary file 6 — Data Management Plan. (DOC 450 kb) [file 13063_2017_1972_MOESM6_ESM.doc]

# DATA MANAGEMENT PLAN

The following **template** can be used to develop a Data Management Plan to accompany a research proposal. The notes (*in italics*) provide further context and guidance for its completion.

If you opt NOT to use the template the topics listed in the template must be addressed.

| **0. Proposal name** | |
| --- | --- |
| **EMPIRIKAL:** An Investigation into the efficacy of Mirococept (APT070) for preventing ischaemia-reperfusion injury in the kidney allograft | |
| **1. Description of the data** | |
| **1.1 Type of study**  This is a multicentre, double-blinded Randomized Controlled phase IIb clinical trial, using an adaptive cumulative cohort design for dose finding and efficacy .  **1.2 Types of data**  Clinical data is recorded in the MACRO database, structured in 22 forms as specified in the following table.   | No. | Forms | D 0 | Baseline | D 1 | D 2 | D 3 | D 4 | D 5 | D 6 | D 7 | D 10 | D 12 | W 2 | W 3 | W 4 | W 5 | W 6 | W 7 | W 8 | W 9 | W 10 | W 11 | W 12 | W 24 | W 36 | W 52 | | --- | --- | --- | --- | --- | --- | --- | --- | --- | --- | --- | --- | --- | --- | --- | --- | --- | --- | --- | --- | --- | --- | --- | --- | --- | --- | --- | | 1 | Registration | x |  |  |  |  |  |  |  |  |  |  |  |  |  |  |  |  |  |  |  |  |  |  |  |  | | 2 | Inclusion/ Exclusion | x |  |  |  |  |  |  |  |  |  |  |  |  |  |  |  |  |  |  |  |  |  |  |  |  | | 3 | Randomisation | x |  |  |  |  |  |  |  |  |  |  |  |  |  |  |  |  |  |  |  |  |  |  |  |  | | 4 | Pre/Post Biospy Sample |  | x |  |  |  |  |  |  |  |  |  |  |  |  |  |  |  |  |  |  |  |  |  |  |  | | 5 | Trial Mediaction Log |  | x |  |  |  |  |  |  |  |  |  |  |  |  |  |  |  |  |  |  |  |  |  |  |  | | 6 | Immunosupresant Therapy |  | x | x | x | x | x | x | x | x | x | x | x | x | x | x | x | x | x | x | x | x | x | x | x | x | | 7 | Recipient Medical History |  | x |  |  |  |  |  |  |  |  |  |  |  |  |  |  |  |  |  |  |  |  |  |  |  | |  | Donor Medical History |  | x |  |  |  |  |  |  |  |  |  |  |  |  |  |  |  |  |  |  |  |  |  |  |  | | 8 | Current Transplant Information |  | x |  |  |  |  |  |  |  |  |  |  |  |  |  |  |  |  |  |  |  |  |  |  |  | | 9 | Base line Vital signs |  | x |  |  |  |  |  |  |  |  |  |  |  |  |  |  |  |  |  |  |  |  |  |  |  | | 10 | Donor History |  | x |  |  |  |  |  |  |  |  |  |  |  |  |  |  |  |  |  |  |  |  |  |  |  | | 11 | Status Form |  |  | x | x | x | x | x | x | x | x | x | x | x | x | x | x | x | x | x | x | x | x | x | x | x | | 12 | Infection Form |  |  | x | x | x | x | x | x | x | x | x | x | x | x | x | x | x | x | x | x | x | x | x | x | x | | 13 | Malignancy Form |  |  | x | x | x | x | x | x | x | x | x | x | x | x | x | x | x | x | x | x | x | x | x | x | x | | 14 | Blood samples |  |  | x | x | x | x | x | x | x | x | x | x | x | x | x | x | x | x | x | x | x | x | x | x | x | | 15 | Research samples |  |  | x | x | x | x | x | x | x | x | x | x | x | x | x | x | x | x | x | x | x | x | x | x | x | | 16 | Month 3 Biopsy Sample |  |  |  |  |  |  |  |  |  |  |  |  |  |  |  |  |  |  |  |  |  | x |  |  |  | | 17 | Adverse Events |  |  |  |  |  |  |  |  |  |  |  |  |  |  |  |  |  |  |  |  |  |  |  |  |  | | 18 | Condmeds |  |  |  |  |  |  |  |  |  |  |  |  |  |  |  |  |  |  |  |  |  |  |  |  |  | | 19 | Graft Failure Form |  |  |  |  |  |  |  |  |  |  |  |  |  |  |  |  |  |  |  |  |  |  |  |  |  | | 20 | Withdrawal Form |  |  |  |  |  |  |  |  |  |  |  |  |  |  |  |  |  |  |  |  |  |  |  |  |  | | 21 | Visit Vital Signs |  |  | x | x | x | x | x | x | x | x | x | x | x | x | x | x | x | x | x | x | x | x | x | x | x | | 22 | 24 Urine Collection |  |  | x |  |  |  |  | x |  |  |  |  |  |  |  |  |  |  |  |  |  |  |  |  |  |   All measurements are numerically coded, unless free text entry is required. Electronic and paper medical records are used as source.  Collection of blood, urine and tissue samples will be tracked via the LIMS database, labsamples.kcl.ac.uk. The raw output of the analysis of these samples will be uploaded and linked to corresponding the LIMS record in its original format (e.g. txt).  Randomisation records are generated and stored by the randomisation system [www.SealedEnvelope.com/empirikal](http://www.SealedEnvelope.com/empirikal).  **1.3 Format and scale of the data**  See Appendix 1 for the codebook and format of the data for this study.  [**X:\Shared\MIROCOCEPT TRIAL\Trial Master File\8. Data Management\8.2 Data manager process & data monitoring plan\EMPIRIKAL_Live_Codebook (2).xls 2014-01-07.xls**](../../../../Volumes/EMPIRIKAL/EMPIRIKAL/Working%20Docs/EMPIRICAL_Live_Codebook%20(2).xls%202014-01-07.xls)  To ensure the long-term validity and storage of the data the KCTU will send a copy of full data export in csv along with the codebook to the CI at the end of the study. Thereafter the data will be the responsibility of the CI and will be archived according to the department SOPs.  *File formats, software used, number of records, databases, sweeps, repetition ns,… (in terms that are meaningful in your field of research). Do formats and software enable sharing and long-term validity of data?* | |
| **2. Data collection / generation** | |
| **2.1 Methodologies for data collection / generation**  *Keep this section concise and accessible to readers who are not data-management experts. Focus on principles, systems and major standards. Focus on the main kind(s) of study data. Give brief examples and avoid long lists.*  An electronic Case Report Form (eCRF) will be created using the InferMed Macro system. Source data will be entered by authorised staff onto the eCRF with a full audit trail. This system is regulatory compliant (GCP, 21CRF11, EC Clinical Trial Directive). The eCRF has been created in collaboration with the Project Manager, trial statistician and the Chief Investigator and maintained by the King’s Clinical Trials Unit. It will be hosted on a dedicated secure server within KCL (Trial Database website address: [www.ctu.co.uk](http://www.ctu.co.uk/) and click the link to MACRO EDC V4).  Appointed research staff (mainly research nurse) from thr recruiting centres will be entering data on the eCRF and responding to raised discrepancies.  **2.2 Data quality and standards**  Inconsistencies in the trial data will be investigated using data queries that prompt the trial centre to clarify or confirm discrepant items. The eCRF system will incorporate automated and manual query generation tools. The Project Manager will systematically check incoming trial data for consistency, omissions and compliance with the protocol. Monitoring of this trial will be to ensure compliance with Good Clinical Practice and scientific integrity will be managed and oversight retained, by the KHP-CTO Quality Team. Details of monitoring are detailed in the Monitoring Plan. Additionally, at every interim, a data export will be sent by the KCTU to the Statistician who will also check the data. At this stage any problems with the data will be reported to the Trial Management Group who will investigate accordingly. | |
| **3. Data management, documentation and curation** | |
| **3.1 Managing, storing and curating data.**  *Briefly, how data will be stored, backed-up, managed and curated in the short to medium term. Specify any community agreed or other formal data standards used (with URL references). [Enter data* security *standards in Section 4].*  The Project Manager will access the data via the EMPIRIKAL _LIVE database and EMPIRIKAL samples LIMS system using the monitoring access setting.  Discrepancies will be flagged on the system for site personnel to address and for monitor to check during site visit.  System back-up will occur daily via the KCL server pathway.  **Planned data Checks**  **The randomization data** export (.csv format) containing Date of Randomisation; site; patient PIN; patient initials and DOB; donation: DBD or DCD status; machine pump perfused organ and kit number will be cross checked against EMPIRIKAL_LIVE:   - Form 1 : registration; DOB, initials - Form 2: exclusion criteria 2 - Form 3 : randomization - Form 5. Trial medication - Form 9: Donor History Q8 & Q15  1. **Primary outcomes status cross check:** Re. Delayed Graft Function (DGF) as patients requiring dialysis in the first week.  - Form 11 status form Q1, Q2, Q4. Q5 and Q6. Q8 and Q9 within 4 weeks of visit time point - Form 12 Infections- Q1 and Q7 - Form 17 AEs - Form 18 Conmeds - Form 19 Graft Failure form - Form 20 withdrawal Q1, Q2 and Q 3  1. Form 6. Immunosupression therapy regime 2. All missing data fields 3. **EMPIRIKAL_LIVE will be cross checked against EMPIRIKAL SAMPLE LIM SYSTEM (**[**labsamples@kcl.ac.uk**](mailto:labsamples@kcl.ac.uk)**)**   Research samples are taken at baseline to week 52 - Was the sample taken Yes / No, date at required time point and location of sample:   - Form 1. PIN, Initials, date of consent, date of transplant (at baseline only) - Status form - Form 16. Month 3 Biopsy Sample Q1. & Q2 - Form 20 Withdrawal Q1, Q2 and Q 3   **3.2 Metadata standards and data documentation**  Any transcription errors between the randomization database and EMPIRIKAL-LIVE should be reported immediately to the Project Manager and trial statistician for discussion with the data managers and the trial personnel in charge of data entry:   1. Discrepancies will be raised for any incomplete data fields within the EMPIRIKAL_LIVE database. 2. Discrepancies will be raised if there are inconsistencies between the forms mentioned in section 3.1 3. List of any time points where samples not taken for analysis (and should have been) or if samples taken erroneously. 4. Data issues detected after the trial statistician’s report to the DMEC at every interim analysis. Especial attention will be taken to monitor proper report of primary outcome, AE, immunosuppressive medication, rejection and infection.   *Plans for documenting, annotating and describing data so that research data are usable by others than your own team. This may include documenting the methods used to generate the data, analytical and procedural information, capturing instrument metadata alongside data, documenting provenance of data and their coding, detailed descriptions for variables, records, etc.*  The CI will receive a copy of the dataset in CSV format on a CD-ROM which will be archived in the TMF from the KCL Clinical Trials Unit at the end of the trial. All trial data will be archived in line with the Medicines for Human Use (Clinical Trials) Amended Regulations 2006 as defined in the KHP-CTO Archiving SOP. | |
| **4. Data security and confidentiality of potentially disclose personal information** | |
| Only anonymised data will be stored on the database are and data entry personnel will ensure patient identifiable data are not included in the text fields of the data entry screens. | |
| **5. Data sharing and access** | |
| Information about the EMPIRIKAL trial and its progress will be available on the project’s website whilst recruitment is open. Results will be publicized to the scientific community by publishing the findings in peer review journals and presenting the data at international and national conferences.  The results and the trial outcome will be disseminated to the lay public by utilising the facilities for communication and education of the public, of the Research Organisation, which is part of the MRC Centre for Transplantation. who’s responsibility it is to provide quality public information on transplant research in conjunction with stakeholders and partners, to promote the research-taking place, and to position the Centre as a Centre of Excellence through the media. | |
| **6. Responsibilities** | |
| The Chief Investigator will act as custodian for the trial data. The KHP-Clinical Research Associate will be responsible for monitoring and source data verification. The Project Manager will remotely check the data listed under section 3.1.  *Specify who, alongside the PI, is responsible for ensuring the study-wide data management, as well as for specific roles such as metadata creation, data security and quality assurance of data.* | |
| **7. Relevant institutional, departmental or study policies on data sharing and data security** | |
| *Please complete, where such policies are (i) relevant to your study, and (ii) are in the public domain, e.g. accessibly through the internet.*  *Add any others that are relevant* | |
| **Policy** | [N/A](http://www.ctu.co.uk/) |
| Data Management Policy & Procedures | In accordance with ICH GCP and KHP-CTO |
| Data Security Policy |  |
| Data Sharing Policy | *e.g. a* [*study policy of sharing research data*](http://www.mrc.ac.uk/Ourresearch/Ethicsresearchguidance/datasharing/Policy/PHSPolicy/studypolicy/index.htm) |
| Institutional Information Policy |  |
| Other: |  |
| Other |  |
| **8. Author of this Data Management Plan (Name)** and, if different to that of the Principal Investigator, their **telephone & email contact details** | |
| Marie Thornhill  **EMPIRIKAL Clinical Project Manager**  Division of Transplantation Immunology & Mucosal Biology, MRC Centre for Transplantation, King's College London, 5th Floor Tower Wing, Guy's Hospital, Great Maze Pond, London, SE1 9RT, Tel: 020 7188 0616, Fax: 020 7188 5660 | |

**Appendix 1: EMPIRIKAL Codebook**

| Title Code | | Question_Status | Code | Name | CAPTION | Type | Format | FIELDORDER |
| --- | --- | --- | --- | --- | --- | --- | --- | --- |
| Title: 24 Hour Urine Collection | | | | | | | | |
|  | UC | 0 | temp_pin | Pin | PIN Number: | Text |  | 1 |
|  | UC | 0 | temp_Initials | temp_Initials | Participant  Initials: | Text |  | 2 |
|  | UC | 0 | UC_01 | UC_01 | 1. Date of sample (dd/mm/yyyy) | Date/Time | dd/mm/yyyy | 3 |
|  | UC | 0 | UC_02 | UC_02 | 2. 24 urine collection (ml) | Integer Number | 9999 | 4 |
| Title: Adverse Events Form | | | | | | | | |
|  | AE | 0 | temp_Initials | temp_Initials | Participant  Initials | Text |  | 1 |
|  | AE | 0 | temp_pin | Pin | PIN Number: | Text |  | 2 |
|  | AE | 0 | AE_00 | AE_00 | Has the participant experienced any Adverse Events since signing the Informed Consent to the trial? | Category |  | 4 |
|  | AE | 0 | AE_09 | AE_09 | SAE Outcome | Category |  | 5 |
|  | AE | 0 | AE_05a | AE_05a | Is Conmed treatment required? | Category |  | 5 |
|  | AE | 0 | AE_01 | AE_01 | Adverse Event   [Diagnosis or symptom  (if known) or signs/ symptoms] | Text |  | 5 |
|  | AE | 0 | AE_02 | AE_02 | Disease related? | Category |  | 5 |
|  | AE | 0 | AE_03 | AE_03 | Body system code | Category |  | 5 |
|  | AE | 0 | AE_04 | AE_04 | Start Date  (dd/mm/yyyy) | Date/Time | dd/mm/yyyy | 5 |
|  | AE | 0 | AE_05 | AE_05 | Stop Date  (dd/mm/yyyy) | Date/Time | dd/mm/yyyy | 5 |
|  | AE | 0 | AE_06 | AE_06 | Intensity | Category |  | 5 |
|  | AE | 0 | AE_07 | AE_07 | Related to  Study drug? | Category |  | 5 |
|  | AE | 0 | AE_08 | AE_08 | Is this a Serious Adverse Event? | Category |  | 5 |
| Title: Blood Results | | | | | | | | |
|  | BR | 0 | temp_pin | Pin | PIN Number: | Text |  | 1 |
|  | BR | 0 | temp_Initials | temp_Initials | Participant  Initials: | Text |  | 2 |
|  | BR | 0 | BR_01 | BR_01 | 1. Date of sample (dd/mm/yyyy) | Date/Time | dd/mm/yyyy | 3 |
|  | BR | 0 | BR_02 | BR_02 | 2. Creatinine (umol/L) | Integer Number | 9999 | 4 |
|  | BR | 0 | BR_03 | BR_03 | 3. eGFR (ml/min) | Integer Number | 999 | 5 |
|  | BR | 0 | BR_04 | BR_04 | 4. Hb (g/L) | Real Number | 99.9 | 6 |
|  | BR | 0 | BR_05 | BR_05 | 5. Tacrolimus trough (ng/ml) Not required at day 1, 3 and 5. | Real Number | 99.99 | 7 |
|  | BR | 0 | BR_05a | BR_05a | 5a. Tacrolimus trough - Time of sampling (hh:mm, 24 hr) | Date/Time | hh:mm | 8 |
|  | BR | 0 | BR_05b | BR_05b | 5b. Tacrolimus trough - Time of dosing (hh:mm, 24 hr) | Date/Time | hh:mm | 9 |
|  | BR | 0 | BR_06 | BR_06 | 6. White Blood Cell Count (WBC) (X 109/L) | Integer Number | 99999 | 10 |
|  | BR | 0 | BR_07 | BR_07 | 7. C- reactive Protein (CRP) (mg/l) | Real Number | 99.99 | 11 |
| Title: Concomitant Medication/Drugs | | | | | | | | |
|  | CM | 0 | temp_pin | Pin | PIN Number: | Text |  | 1 |
|  | CM | 0 | temp_Initials | temp_Initials | Participant  Initials: | Text |  | 2 |
|  | CM | 0 | CM_00 | CM_00 | Is this medication  disease related? | Category |  | 3 |
|  | CM | 0 | CM_01 | CM_01 | Name of Medication  (Brand or Generic) | Text |  | 3 |
|  | CM | 0 | CM_02 | CM_02 | Body System code | Category |  | 3 |
|  | CM | 0 | CM_03 | CM_03 | Date Started  (dd/mm/yy) | Date/Time | dd/mm/yyyy | 3 |
|  | CM | 0 | CM_04 | CM_04 | Date Stopped  (dd/mm/yy) | Date/Time | dd/mm/yyyy | 3 |
|  | CM | 0 | CM_05 | CM_05 | Dose | Real Number | 999.99 | 3 |
|  | CM | 0 | CM_06 | CM_06 | Units | Category |  | 3 |
|  | CM | 0 | CM_07 | CM_07 | Frequency | Category |  | 3 |
|  | CM | 0 | CM_08 | CM_08 | Continuing at end of study (Complete at Month 12 or  withdrawal) | Category |  | 3 |
| Title: Current Transplant Information | | | | | | | | |
|  | CT | 0 | temp_pin | Pin | PIN Number: | Text |  | 1 |
|  | CT | 0 | temp_Initials | temp_Initials | Participant  Initials: | Text |  | 2 |
|  | CT | 0 | CT_01a | CT_01a | 1a. A | Integer Number | 99 | 3 |
|  | CT | 0 | CT_01b | CT_01b | 1b. B | Integer Number | 99 | 4 |
|  | CT | 0 | CT_01c | CT_01c | 1c. DR | Integer Number | 99 | 5 |
|  | CT | 0 | CT_02 | CT_02 | 2. Calculated reaction frequency (CRF) (%) | Integer Number | 999 | 6 |
|  | CT | 0 | CT_03 | CT_03 | 3. Donor Specific Antibodies (DSA) | Category |  | 7 |
|  | CT | 0 | CT_03a | CT_03a | 3a. DSA Classification | Category |  | 8 |
| Title: Donor History | | | | | | | | |
|  | DH | 0 | temp_pin | Pin | PIN Number: | Text |  | 1 |
|  | DH | 0 | temp_Initials | temp_Initials | Participant  Initials: | Text |  | 2 |
|  | DH | 0 | DH_01 | DH_01 | 1. NHSBT Donor ID (numerical field of 7 digits) | Integer Number | 9999999 | 3 |
|  | DH | 0 | DH_02 | DH_02 | 2. Donor date of birth (dd/mm/yyyy) | Date/Time | dd/mm/yyyy | 4 |
|  | DH | 0 | DH_03 | DH_03 | 3. Donor sex | Category |  | 5 |
|  | DH | 0 | DH_04 | DH_04 | 4. Ethnicity | Category |  | 6 |
|  | DH | 0 | DH_05 | DH_05 | 5. Height (cm) | Integer Number | 999 | 7 |
|  | DH | 0 | DH_06 | DH_06 | 6. Weight (kg) | Integer Number | 999 | 8 |
|  | DH | 0 | DH_07 | DH_07 | 7. Cause of death | Category |  | 9 |
|  | DH | 0 | DH_07a | DH_07a | 7a. Cause of death - Stroke | Category |  | 10 |
|  | DH | 0 | DH_08 | DH_08 | 8. Type of donation | Category |  | 11 |
|  | DH | 0 | DH_08a | DH_08a | 8a. Maastricht classification for DCD donor | Category |  | 12 |
|  | DH | 0 | DH_09 | DH_09 | 9. Time of withdrawal of life support? (hh:mm, 24 hr) | Date/Time | hh:mm | 13 |
|  | DH | 0 | DH_10 | DH_10 | 10. Date of death (dd/mm/yyyy) | Date/Time | dd/mm/yyyy | 14 |
|  | DH | 0 | DH_10a | DH_10a | 10a. Time of death? (hh:mm, 24 hr) | Date/Time | hh:mm | 15 |
|  | DH | 0 | DH_11 | DH_11 | 11. Date of perfusion of organ at retrieval? (dd/mm/yyyy) | Date/Time | dd/mm/yyyy | 16 |
|  | DH | 0 | DH_11a | DH_11a | 11a. Time of perfusion at organ retrieval? (hh:mm, 24 hr) | Date/Time | hh:mm | 17 |
|  | DH | 0 | DH_12 | DH_12 | 12. Date organ put on ice (dd/mm/yyyy) | Date/Time | dd/mm/yyyy | 18 |
|  | DH | 0 | DH_12a | DH_12a | 12a. Time of organ put on ice? (hh:mm, 24 hr) | Date/Time | hh:mm | 19 |
|  | DH | 0 | DH_13 | DH_13 | 13. Date organ taken off ice (dd/mm/yyyy) | Date/Time | dd/mm/yyyy | 20 |
|  | DH | 0 | DH_13a | DH_13a | 13a. Time of organ taken off ice? (hh:mm, 24 hr) | Date/Time | hh:mm | 21 |
|  | DH | 0 | DH_14 | DH_14 | 14. Date of perfusion with recipient blood? (dd/mm/yyyy) | Date/Time | dd/mm/yyyy | 22 |
|  | DH | 0 | DH_14a | DH_14a | 14a. Time of perfusion with recipient blood? (hh:mm, 24 hr) | Date/Time | hh:mm | 23 |
|  | DH | 0 | DH_15 | DH_15 | 15. Machine pump use | Category |  | 24 |
|  | DH | 0 | DH_16 | DH_16 | 16. Donor Blood culture | Category |  | 25 |
|  | DH | 0 | DH_17 | DH_17 | 17. Donor urine Culture | Category |  | 26 |
|  | DH | 0 | DH_18 | DH_18 | 18. Donor serum creatinine at time of death (µmol/L) | Integer Number | 999 | 27 |
|  | DH | 0 | DH_19 | DH_19 | 19. Donor serum creatinine on admission (µmol/L) | Integer Number | 999 | 28 |
| Title: Eligibility Form | | | | | | | | |
|  | ELIG | 0 | temp_pin | Pin | PIN Number: | Text |  | 3 |
|  | ELIG | 0 | temp_Initials | temp_Initials | Participant  Initials: | Text |  | 4 |
|  | ELIG | 0 | IN_01 | IN_01 | 1. Patient must be 16 years of age or older and registered on the kidney | Category |  | 6 |
|  | ELIG | 0 | IN_02 | IN_02 | 2. Patient must be willing to participate in the study and provide written | Category |  | 7 |
|  | ELIG | 0 | IN_03 | IN_03 | 3. Patient must have the ability to comply with the study requirements. | Category |  | 8 |
|  | ELIG | 0 | IN_04 | IN_04 | 4. Donor must be older than 10 years of age. | Category |  | 14 |
|  | ELIG | 0 | IN_05 | IN_05 | 5. Patient is on dialysis. | Category |  | 15 |
|  | ELIG | 0 | EX_01 | EX_01 | 1. Patient is recipient of a living-donor kidney. | Category |  | 16 |
|  | ELIG | 0 | EX_02 | EX_02 | 2. Patient is a recipient of a DCD kidney Maastricht category 1 or 2. | Category |  | 17 |
|  | ELIG | 0 | EX_03 | EX_03 | 3. Patient has evidence of current or previous infection of HIV, HBV or HCV. | Category |  | 18 |
|  | ELIG | 0 | EX_04 | EX_04 | 4. Patient is recipient of an en bloc paediatric or an adult double renal transplant. | Category |  | 19 |
|  | ELIG | 0 | EX_05 | EX_05 | 5. The donor kidney has more than 2 renal arteries, unless the artery is small | Category |  | 20 |
|  | ELIG | 0 | EX_06 | EX_06 | 6. Any ABO blood group or HLA incompatible transplant. | Category |  | 21 |
|  | ELIG | 0 | EX_07 | EX_07 | 7. Patients receiving donor organs with a cold ischaemic time >30 hours before  treatment with Mirococept. | Category |  | 22 |
|  | ELIG | 0 | EX_08 | EX_08 | 8. Any recipient of a multi-organ transplant or a previous recipient of a non-renal | Category |  | 23 |
|  | ELIG | 0 | EX_09 | EX_09 | 9. Females who are pregnant or lactating. | Category |  | 24 |
|  | ELIG | 0 | EX_10 | EX_10 | 10. Patients not willing to use contraception for at least one month post transplant. | Category |  | 25 |
|  | ELIG | 0 | EX_11 | EX_11 | 11. Patients with a history of malignancy within the last 5 years, except adequately | Category |  | 26 |
|  | ELIG | 0 | EX_12 | EX_12 | 12. Patients prescribed Antithymocyte globulin (ATG) or Alemtuzumab (Campath) | Category |  | 27 |
|  | ELIG | 0 | EX_13 | EX_13 | 13. Patients involved in other experimental drug trials. | Category |  | 28 |
| Title: Graft Failure Form | | | | | | | | |
|  | GF | 0 | temp_pin | Pin | PIN Number: | Text |  | 1 |
|  | GF | 0 | temp_Initials | temp_Initials | Participant  Initials: | Text |  | 2 |
|  | GF | 0 | GF_01 | GF_01 | 1. Has transplant failed? | Category |  | 3 |
|  | GF | 0 | GF_01a | GF_01a | 1a. Main cause of graft failure | Category |  | 4 |
|  | GF | 0 | GF_02 | GF_02 | 2. Definition of transplantation failure | Category |  | 5 |
|  | GF | 0 | GF_02a | GF_02a | 2a. Date of transplant failure (ie date of initiation of | Date/Time | dd/mm/yyyy | 6 |
| Title: Hospitalisation Log | | | | | | | | |
|  | HOS | 0 | temp_pin | Pin | PIN Number: | Text |  | 1 |
|  | HOS | 0 | temp_Initials | temp_Initials | Participant  Initials: | Text |  | 2 |
|  | HOS | 0 | HOS_01 | HOS_01 | 1. Date of discharge following transplant? (dd/mm/yyyy) | Date/Time | dd/mm/yyyy | 3 |
|  | HOS | 0 | HOS_02 | HOS_02 | 2. Has the participant been hospitalised since transplant? | Category |  | 4 |
|  | HOS | 0 | HOS_03a | HOS_03a | Admission Date  (dd/mm/yy) | Date/Time | dd/mm/yyyy | 5 |
|  | HOS | 0 | HOS_03b | HOS_03b | Discharge Date  (dd/mm/yy) | Date/Time | dd/mm/yyyy | 5 |
|  | HOS | 0 | HOS_03c | HOS_03c | Reason for Admission? | Text |  | 5 |
| Title: Immunosuppression Therapy | | | | | | | | |
|  | IMM | 0 | temp_pin | Pin | PIN Number: | Text |  | 1 |
|  | IMM | 0 | temp_Initials | temp_Initials | Participant  Initials: | Text |  | 2 |
|  | IMM | 0 | IMM_01 | IMM_01 | 1. Start date of dose (dd/mm/yyyy) | Date/Time | dd/mm/yyyy | 3 |
|  | IMM | 0 | IMM_02a | IMM_02a | 2a. Tacrolimus | Category |  | 4 |
|  | IMM | 0 | IMM_02b | IMM_02b | 2b. Tacrolimus Dose (mg) | Real Number | 999.99 | 5 |
|  | IMM | 0 | IMM_02c | IMM_02c | 2c. Tacrolimus Frequency | Category |  | 6 |
|  | IMM | 0 | IMM_03a | IMM_03a | 3a. Mycophenolate Mofetil | Category |  | 7 |
|  | IMM | 0 | IMM_03b | IMM_03b | 3b. Mycophenolate Mofetil Dose (mg) | Real Number | 9999.99 | 8 |
|  | IMM | 0 | IMM_03c | IMM_03c | 3c. Mycophenolate Mofetil Frequency | Category |  | 9 |
|  | IMM | 0 | IMM_04a | IMM_04a | 4a. Prednisolone | Category |  | 10 |
|  | IMM | 0 | IMM_04b | IMM_04b | 4b. Prednisolone Dose (mg) | Integer Number | 999 | 11 |
|  | IMM | 0 | IMM_04c | IMM_04c | 4c. Prednisolone Frequency | Category |  | 12 |
|  | IMM | 0 | IMM_05a | IMM_05a | 5a. Azathioprine | Category |  | 13 |
|  | IMM | 0 | IMM_05b | IMM_05b | 5b. Azathioprine Dose (mg) | Real Number | 99.9 | 14 |
|  | IMM | 0 | IMM_05c | IMM_05c | 5c. Azathioprine Frequency | Category |  | 15 |
|  | IMM | 0 | IMM_06a | IMM_06a | 6a. Cyclosporine | Category |  | 16 |
|  | IMM | 0 | IMM_06b | IMM_06b | 6b. Cyclosporine Dose (mg) | Real Number | 99.99 | 17 |
|  | IMM | 0 | IMM_06c | IMM_06c | 6c. Cyclosporine Frequency | Category |  | 18 |
|  | IMM | 0 | IMM_07a | IMM_07a | 7a. Other | Category |  | 19 |
|  | IMM | 0 | IMM_07b | IMM_07b | 7b. Other Dose (mg) | Text |  | 20 |
|  | IMM | 0 | IMM_07c | IMM_07c | 7c. Other Frequency | Category |  | 21 |
| Title: Infection Form | | | | | | | | |
|  | INF | 0 | temp_pin | Pin | PIN Number: | Text |  | 1 |
|  | INF | 0 | temp_Initials | temp_Initials | Participant  Initials: | Text |  | 2 |
|  | INF | 0 | INF_01 | INF_01 | 1. Did the patient develop a new infection or infections since the last time point? | Category |  | 3 |
|  | INF | 0 | INF_02 | INF_02 | 2. Has the patient reported a viral infection? | Category |  | 4 |
|  | INF | 0 | INF_03 | INF_03 | 3. Was the viral infection confirmed by culture/PCR? | Category |  | 5 |
|  | INF | 0 | INF_04 | INF_04 | 4. Was the viral infection confirmed as BK? | Category |  | 6 |
|  | INF | 0 | INF_05 | INF_05 | 5. Was the viral infection confirmed as EBV? | Category |  | 7 |
|  | INF | 0 | INF_06 | INF_06 | 6. Was the viral infection confirmed as herpes zoster virus? | Category |  | 8 |
|  | INF | 0 | INF_07 | INF_07 | 7. Has patient had any cytomegalovirus (CMV)? | Category |  | 9 |
|  | INF | 0 | INF_08 | INF_08 | 8. Has patient had any positive UTIs? | Category |  | 10 |
|  | INF | 0 | INF_09 | INF_09 | 9. Has the patient reported a bacterial infection? | Category |  | 11 |
|  | INF | 0 | INF_10 | INF_10 | 10. Was the bacterial infection confirmed by culture/PCR or by appropriate imaging? | Category |  | 12 |
|  | INF | 0 | INF_11 | INF_11 | 11. Was the bacterial infection confirmed as a pneumonia? | Category |  | 13 |
|  | INF | 0 | INF_12 | INF_12 | 12. Was the bacterial infection confirmed as TB? | Category |  | 14 |
|  | INF | 0 | INF_13 | INF_13 | 13. FUNGAL - Has the patient reported a fungal infection? | Category |  | 15 |
|  | INF | 0 | INF_14 | INF_14 | 14. Was the fungal infection confirmed as pneumocystis jirovecii? | Category |  | 16 |
| Title: Malignancy Form | | | | | | | | |
|  | MAL | 0 | temp_pin | Pin | PIN Number: | Text |  | 1 |
|  | MAL | 0 | temp_Initials | temp_Initials | Participant  Initials: | Text |  | 2 |
|  | MAL | 0 | MAL_01 | MAL_01 | 1. Did the patient develop a new malignancy since the last visit? | Category |  | 3 |
|  | MAL | 0 | MAL_02 | MAL_02 | 2. Was it confirmed histologically? | Category |  | 4 |
|  | MAL | 0 | MAL_03 | MAL_03 | 3. What was the site of the primary malignancy? | Category |  | 5 |
|  | MAL | 0 | MAL_03a | MAL_03a | 3a. Other, please specify | Text |  | 6 |
|  | MAL | 0 | MAL_04 | MAL_04 | 4. Is malignancy confirmed to be related to a viral infection? | Category |  | 7 |
| Title: Medical History | | | | | | | | |
|  | MEDH | 0 | temp_pin | Pin | PIN Number: | Text |  | 1 |
|  | MEDH | 0 | temp_Initials | temp_Initials | Participant  Initials: | Text |  | 2 |
|  | MEDH | 0 | MEDH_01 | MEDH_01 | 1. Cardiovascular | Category |  | 4 |
|  | MEDH | 0 | MEDH_02 | MEDH_02 | 2. Respiratory | Category |  | 5 |
|  | MEDH | 0 | MEDH_03 | MEDH_03 | 3. Hepatic | Category |  | 6 |
|  | MEDH | 0 | MEDH_04 | MEDH_04 | 4. Gastro-intestinal | Category |  | 7 |
|  | MEDH | 0 | MEDH_05 | MEDH_05 | 5. Genito-urinary | Category |  | 8 |
|  | MEDH | 0 | MEDH_06 | MEDH_06 | 6. Endocrine | Category |  | 9 |
|  | MEDH | 0 | MEDH_07 | MEDH_07 | 7. Haematological | Category |  | 10 |
|  | MEDH | 0 | MEDH_08 | MEDH_08 | 8. Musculo-skeletal | Category |  | 11 |
|  | MEDH | 0 | MEDH_09 | MEDH_09 | 9. Neoplasia | Category |  | 12 |
|  | MEDH | 0 | MEDH_10 | MEDH_10 | 10. Neurological | Category |  | 13 |
|  | MEDH | 0 | MEDH_11 | MEDH_11 | 11. Psychiatric | Category |  | 14 |
|  | MEDH | 0 | MEDH_12 | MEDH_12 | 12. Immunological | Category |  | 15 |
|  | MEDH | 0 | MEDH_13 | MEDH_13 | 13. Dermatological | Category |  | 16 |
|  | MEDH | 0 | MEDH_14 | MEDH_14 | 14. Allergies | Category |  | 17 |
|  | MEDH | 0 | MEDH_15 | MEDH_15 | 15. Eyes, ear, nose, throat | Category |  | 18 |
|  | MEDH | 0 | MEDH_16 | MEDH_16 | 16. Other | Category |  | 19 |
|  | MEDH | 0 | MEDH_17 | MEDH_17 | Code: | Category |  | 42 |
|  | MEDH | 0 | MEDH_18 | MEDH_18 | Medical Condition | Text |  | 42 |
|  | MEDH | 0 | MEDH_19 | MEDH_19 | Dates | Text |  | 42 |
|  | MEDH | 0 | MEDH_20 | MEDH_20 | Currently ongoing? | Category |  | 42 |
|  | MEDH | 0 | MEDH_21 | MEDH_21 | 21. Cause of primary kidney failure | Category |  | 43 |
|  | MEDH | 0 | MEDH_22a | MEDH_22a | 22a. Previous number of transplants? | Integer Number | 99 | 44 |
|  | MEDH | 0 | MEDH_22b | MEDH_22b | 22b. Month & year (mm/yyyy) | Date/Time | dd/mm/yyyy | 45 |
|  | MEDH | 0 | MEDH_22c | MEDH_22c | 22c. Month & year (mm/yyyy) | Date/Time | dd/mm/yyyy | 46 |
|  | MEDH | 0 | MEDH_22d | MEDH_22d | 22d. Month & year (mm/yyyy) | Date/Time | dd/mm/yyyy | 47 |
|  | MEDH | 0 | MEDH_22e | MEDH_22e | 22e. Month & year (mm/yyyy) | Date/Time | dd/mm/yyyy | 48 |
|  | MEDH | 0 | MEDH_23 | MEDH_23 | 23. Current Mode of dialysis? | Category |  | 49 |
|  | MEDH | 0 | MEDH_24 | MEDH_24 | 24. Start date for current mode dialysis? (mm/yyyy) | Date/Time | dd/mm/yyyy | 50 |
|  | MEDH | 0 | MEDH_25 | MEDH_25 | 25. Cause of most recent graft failure(s ) | Category |  | 51 |
| Title: Month 3 Biopsy Sample | | | | | | | | |
|  | BS | 0 | temp_pin | Pin | PIN Number: | Text |  | 1 |
|  | BS | 0 | temp_Initials | temp_Initials | Participant  Initials: | Text |  | 2 |
|  | BS | 0 | BS_01 | BS_01 | 1. Has patient consented to Month 3 biopsy? | Category |  | 3 |
|  | BS | 0 | BS_02 | BS_02 | 2. Date of sample taken (dd/mm/yyyy) | Date/Time | dd/mm/yyyy | 4 |
| Title: PI End of study sign off | | | | | | | | |
|  | SO | 0 | temp_pin | Pin | PIN Number: | Text |  | 1 |
|  | SO | 0 | temp_Initials | temp_Initials | Participant  Initials: | Text |  | 2 |
|  | SO | 0 | SO_01 | SO_01 | 1. PI Signoff of Participant data: | Category |  | 4 |
| Title: Pre/Post Perfusion Biopsy Samples | | | | | | | | |
|  | BIOP | 0 | temp_pin | Pin | PIN Number: | Text |  | 1 |
|  | BIOP | 0 | temp_Initials | temp_Initials | Participant  Initials: | Text |  | 2 |
|  | BIOP | 0 | BIOP_00 | BIOP_00 | 0. Date of sample taken (dd/mm/yyyy): | Date/Time | dd/mm/yyyy | 4 |
| Title: Randomisation Form | | | | | | | | |
|  | RAN | 0 | temp_pin | Pin | PIN Number: | Text |  | 1 |
|  | RAN | 0 | temp_Initials | temp_Initials | Participant  Initials: | Text |  | 2 |
|  | RAN | 0 | RAN_01 | RAN_01 | 1. Date randomised (dd/mm/yyyy): | Date/Time | dd/mm/yyyy | 3 |
| Title: Registration Form | | | | | | | | |
|  | REG | 0 | REG_01 | REG_01 | 1. Participant initials | Text |  | 1 |
|  | REG | 0 | REG_02 | REG_02 | 2. Participant date of birth (dd/mm/yyyy) | Date/Time | dd/mm/yyyy | 2 |
|  | REG | 0 | REG_03 | REG_03 | 3. Participant sex | Category |  | 3 |
|  | REG | 0 | REG_04 | REG_04 | 4. Ethnicity | Category |  | 4 |
|  | REG | 0 | REG_05 | REG_05 | 5. Date of consent (dd/mm/yyyy) | Date/Time | dd/mm/yyyy | 5 |
|  | REG | 1 | REG_06 | REG_06 | Site | Text |  | 6 |
| Title: Research Samples | | | | | | | | |
|  | RS | 0 | temp_pin | Pin | PIN Number: | Text |  | 1 |
|  | RS | 0 | temp_Initials | temp_Initials | Participant  Initials: | Text |  | 2 |
|  | RS | 0 | RS_01 | RS_01 | 1. Date of sample (dd/mm/yyyy) | Date/Time | dd/mm/yyyy | 3 |
|  | RS | 0 | RS_02a | RS_02a | 2a. Mirococept & Serum for antibodies to Mirococept determination | Category |  | 4 |
|  | RS | 0 | RS_02b | RS_02b | 2b. Serum complement levels (CH50%) | Category |  | 5 |
|  | RS | 0 | RS_02c | RS_02c | 2c. Urine for markers of tubular damage - RBP, NAG, NGAL, | Category |  | 6 |
|  | RS | 0 | RS_02d | RS_02d | 2d. Urine for Bio-markers (within 4 hours of production) - | Category |  | 7 |
|  | RS | 0 | RS_02e | RS_02e | 2e. Whole Blood for bio-marker (Cohort one only) | Category |  | 8 |
|  | RS | 0 | RS_02f | RS_02f | 2f. Urine C3a | Category |  | 9 |
|  | RS | 0 | RS_02g | RS_02g | 2g. Blood plasma (Li Heparin) SDMA (symmetric dimethyl arginine) -1/2 ml | Category |  | 10 |
| Title: Status Form | | | | | | | | |
|  | STA | 0 | temp_pin | Pin | PIN Number: | Text |  | 1 |
|  | STA | 0 | temp_Initials | temp_Initials | Participant  Initials: | Text |  | 2 |
|  | STA | 0 | STA_01 | STA_01 | 1. Current status: Where necessary please complete a withdrawl form. | Category |  | 3 |
|  | STA | 0 | STA_02 | STA_02 | 2. Date of death (dd/mm/yyyy) | Date/Time | dd/mm/yyyy | 4 |
|  | STA | 0 | STA_03 | STA_03 | 3. Cause of death | Category |  | 5 |
|  | STA | 0 | STA_04 | STA_04 | 4. Has patient refused data collection at this time-point? | Category |  | 6 |
|  | STA | 0 | STA_05 | STA_05 | 5. Has the patient had post transplant dialysis? | Category |  | 7 |
|  | STA | 0 | STA_05a | STA_05a | 5a. Date of last dialysis session (dd/mm/yyyy) | Date/Time | dd/mm/yyyy | 8 |
|  | STA | 0 | STA_06 | STA_06 | 6. Mode of dialysis | Category |  | 9 |
|  | STA | 0 | STA_07 | STA_07 | 7. Has patient had any episode of Calcineurin Inhibitor Nephrotoxicity | Category |  | 10 |
|  | STA | 0 | STA_08 | STA_08 | 8. Has patient had any new episodes of acute rejection since the last time point ? | Category |  | 11 |
|  | STA | 0 | STA_09 | STA_09 | 9. Was the new rejection episode confirmed by biopsy? | Category |  | 12 |
| Title: Trial Medication Log (Mirococept or Placebo) | | | | | | | | |
|  | TML | 0 | temp_pin | Pin | PIN Number: | Text |  | 1 |
|  | TML | 0 | temp_Initials | temp_Initials | Participant  Initials: | Text |  | 2 |
|  | TML | 0 | TML_01 | TML_01 | 1. Drug Kit Number | Text | AA99 | 3 |
|  | TML | 0 | TML_02 | TML_02 | 2. Date of perfusion (dd/mm/yyyy) | Date/Time | dd/mm/yyyy | 4 |
|  | TML | 0 | TML_03 | TML_03 | 3. Time of perfusion: hh:mm | Date/Time | hh:mm | 5 |
| Title: vdate | | | | | | | | |
|  | vdate | 0 | visit_date | visit_date | visit_date | Date/Time | dd/mm/yyyy | 1 |
| Title: Visit Vital Signs | | | | | | | | |
|  | VS | 0 | temp_pin | Pin | PIN Number: | Text |  | 1 |
|  | VS | 0 | temp_Initials | temp_Initials | Participant  Initials: | Text |  | 2 |
|  | VS | 0 | VS_01 | VS_01 | 1. Temperature (°C) | Integer Number | 999 | 3 |
|  | VS | 0 | VS_02 | VS_02 | 2. Pulse rate (bpm) | Integer Number | 999 | 4 |
|  | VS | 0 | VS_03a | VS_03a |  | Integer Number | 999 | 5 |
|  | VS | 0 | VS_03b | VS_03b |  | Integer Number | 999 | 6 |
|  | VS | 0 | VS_04 | VS_04 | 4. Weight (Kg) | Integer Number | 999 | 7 |
| Title: Vital Signs | | | | | | | | |
|  | VIT | 0 | temp_pin | Pin | PIN Number: | Text |  | 1 |
|  | VIT | 0 | temp_Initials | temp_Initials | Participant  Initials: | Text |  | 2 |
|  | VIT | 0 | VIT_01 | VIT_01 | 1. Temperature (°C) | Integer Number | 999 | 3 |
|  | VIT | 0 | VIT_02 | VIT_02 | 2. Pulse rate (bpm) | Integer Number | 999 | 4 |
|  | VIT | 0 | VIT_03a | VIT_03a | 3a. Blood pressure | Integer Number | 999 | 5 |
|  | VIT | 0 | VIT_03b | VIT_03b | 3b. Blood pressure | Integer Number | 999 | 6 |
|  | VIT | 0 | VIT_04 | VIT_04 | 4. Height (cm) | Integer Number | 999 | 7 |
|  | VIT | 0 | VIT_05 | VIT_05 | 5. Weight (kg) | Integer Number | 999 | 8 |
| Title: Withdrawal Form | | | | | | | | |
|  | WD | 0 | temp_pin | Pin | PIN Number: | Text |  | 1 |
|  | WD | 0 | temp_Initials | temp_Initials | Participant  Initials: | Text |  | 2 |
|  | WD | 0 | WD_01 | WD_01 | 1. Has the participant withdrawn from follow-up? | Category |  | 4 |
|  | WD | 0 | WD_02 | WD_02 | 2. Date of withdrawal (dd/mm/yyyy) | Date/Time | dd/mm/yyyy | 5 |
|  | WD | 0 | WD_03 | WD_03 | 3. Reason for withdrawal | Category |  | 6 |
|  | WD | 0 | WD_03a | WD_03a | 3a. If other, please specify reason for withdrawal | Text |  | 7 |
|  | WD | 0 | WD_04 | WD_04 | 4. Briefly describe the circumstances of the withdrawal | Text |  | 9 |
|  | WD | 0 | WD_05 | WD_05 | 5. Withdrawal decision initiated by | Category |  | 10 |
|  | WD | 0 | WD_05a | WD_05a | 5a. If other, please specify who initiated the withdrawal decision | Text |  | 11 |
| Title: z. Template | | | | | | | | |
|  | Template | 0 | temp_pin | Pin | PIN Number: | Text |  | 1 |
|  | Template | 0 | temp_Initials | temp_Initials | Participant  Initials: | Text |  | 2 |
